# Halide perovskites as disposable epitaxial templates for the phase-selective synthesis of lead sulfochloride nanocrystals

**DOI:** 10.1038/s41467-022-31699-1

**Published:** 2022-07-08

**Authors:** Stefano Toso, Muhammad Imran, Enrico Mugnaioli, Anna Moliterni, Rocco Caliandro, Nadine J. Schrenker, Andrea Pianetti, Juliette Zito, Francesco Zaccaria, Ye Wu, Mauro Gemmi, Cinzia Giannini, Sergio Brovelli, Ivan Infante, Sara Bals, Liberato Manna

**Affiliations:** 1 Department of Nanochemistry, Istituto Italiano di Tecnologia, Via Morego 30, 16163 Genova, Italy; 2 International Doctoral Program in Science, Università Cattolica del Sacro Cuore, 25121 Brescia, Italy; 3 Electron Crystallography, Center for Materials Interfaces, Istituto Italiano di Tecnologia, Viale Rinaldo Piaggio 34, 56025 Pontedera, Italy; 4 Istituto di Cristallografia – Consiglio Nazionale delle Ricerche (IC–CNR), Via Giovanni Amendola 122/O, 70126 Bari, Italy; 5 Electron Microscopy for Materials Science (EMAT) and NANOlab Center of Excellence, University of Antwerp, Groenenborgerlaan 171, 2020 Antwerp, Belgium; 6 Dipartimento di Scienza dei Materiali, Università degli Studi di Milano-Bicocca, Via Roberto Cozzi 55, 20125 Milano, Italy; 7 Dipartimento di Chimica e Chimica Industriale, Università degli Studi di Genova, Via Dodecaneso 31, 16146 Genova, Italy

**Keywords:** Nanoparticles, Nanoparticles

## Abstract

Colloidal chemistry grants access to a wealth of materials through simple and mild reactions. However, even few elements can combine in a variety of stoichiometries and structures, potentially resulting in impurities or even wrong products. Similar issues have been long addressed in organic chemistry by using reaction-directing groups, that are added to a substrate to promote a specific product and are later removed. Inspired by such approach, we demonstrate the use of CsPbCl_3_ perovskite nanocrystals to drive the phase-selective synthesis of two yet unexplored lead sulfochlorides: Pb_3_S_2_Cl_2_ and Pb_4_S_3_Cl_2_. When homogeneously nucleated in solution, lead sulfochlorides form Pb_3_S_2_Cl_2_ nanocrystals. Conversely, the presence of CsPbCl_3_ triggers the formation of Pb_4_S_3_Cl_2_/CsPbCl_3_ epitaxial heterostructures. The phase selectivity is guaranteed by the continuity of the cationic subnetwork across the interface, a condition not met in a hypothetical Pb_3_S_2_Cl_2_/CsPbCl_3_ heterostructure. The perovskite domain is then etched, delivering phase-pure Pb_4_S_3_Cl_2_ nanocrystals that could not be synthesized directly.

## Abstract (web summary)

Phase-selective approaches, such using reaction-directing groups, are often seen in traditional organic chemistry and catalysis. Here authors use perovskite nanocrystals as disposable templates to drive the phase-selective synthesis of two colloidal nanomaterials, the lead sulfohalides Pb_3_S_2_Cl_2_ and Pb_4_S_3_Cl_2_.

## Introduction

Since its early days^1^, the research on colloidal nanocrystals (NCs) has provided access to a variety of inorganic compounds, spanning from metals and alloys^2^ to oxides^3,4^, phosphides^5^, chalcogenides^6–11^, halides^12,13^, and more. Nowadays, NCs are exploited in many different fields, as NCs-based products are steadily finding their way to commercial applications^14–18^. A colloidal approach to the synthesis of inorganic materials offers several advantages over more traditional solid-state chemistry techniques, such as milder reaction conditions, ease of tunability deriving from a wider choice of precursors, better processability of the products, and straightforward access to nanostructured materials. However, both this flexibility and the push toward materials combining a higher number of elements come at the cost of increased complexity. One example is the field of colloidal semiconductors, where lead-based NCs are among the most explored compounds due to the appealing optoelectronic properties demonstrated by lead halide perovskites in the UV-VIS and by lead chalcogenides in the IR spectral ranges^6,7,12,13,19^. There, the combination of as little as four elements (Cs, Pb, X and E, where X = F, Cl, Br or I, and E = S, Se or Te) can yield NCs of a variety of phases: the binaries CsX, PbX_2_, and PbE^6,7,20–22^, the well-known cesium lead halides (Cs_4_PbX_6_, CsPbX_3_, and CsPb_2_X_5_)^12,19,23,24^, and the still little explored lead chalcohalides (Pb_4_S_3_Br_2_ and Pb_4_S_3_I_2_)^25,26^. All these compounds are often obtained under similar reaction conditions, and can therefore compete during the synthesis, requiring a careful tuning of the synthetic protocols to achieve an impurity-free synthesis of the desired product.

This condition is not dissimilar from that of many organic chemistry reactions, where two molecules can react following different pathways and resulting in different products. Similar issues are often addressed by exploiting reaction-directing groups, that are moieties which are attached to one of the reagents in order to direct the reaction pathway toward the desired product, and are removed at a later stage^27^. This requires a multi-step rational design of the synthetic procedure, that is common for organic chemistry but is missing in the colloidal synthesis of NCs, as most procedures consist of a one-step reaction.

**Fig. 1:**
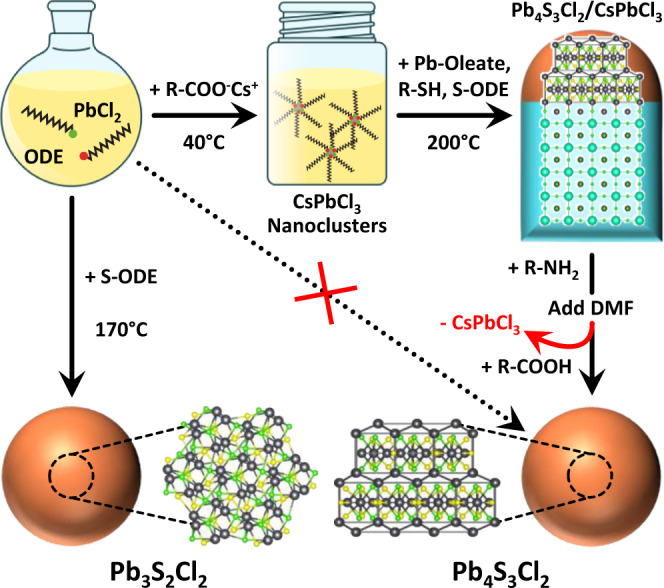
Phase-selective templated synthesis of lead sulfochloride NCs. A solution of PbCl_2_ reacts with sulfur dissolved in 1-octadecene (S-ODE) to form Pb_3_S_2_Cl_2_ NCs. When reacted with Cs-oleate (R-COO^-^Cs^+^) it produces instead CsPbCl_3_ nanoclusters, that can be further reacted with Pb-oleate, S-ODE, and an alkyl thiol (R-SH) to form Pb_4_S_3_Cl_2_/CsPbCl_3_ heterostructures. The CsPbCl_3_ domain can be then selectively etched by the sequential addition of oleylamine (R-NH_2_), dimethyl formamide (DMF), and oleic acid (R-COOH), yielding colloidally stable Pb_4_S_3_Cl_2_ NCs that could not be obtained by direct synthesis. Atoms color code: Cs = cyan; Pb = gray; S = yellow; Cl = green.

In this work, we exploit the formation of epitaxial heterostructures as a reaction-directing step for the phase-selective synthesis of colloidal NCs, and we take advantage of this approach to further expand the family of lead chalcohalides. These materials share the general formula Pb_*a*_E_*b*_X_*c*_, and only four stable compositions are known in bulk (PbTeF_6_, Pb_7_S_2_Br_10_, Pb_5_S_2_I_6_, Pb_4_SeBr_6_)^28,29^, together with two recently reported high-pressure metastable phases (Pb_4_S_3_I_2_, Pb_3_Se_2_Br_2_)^30,31^. Our group pioneered the investigation of lead chalcohalides at the nanoscale, discovering two additional compounds (Pb_4_S_3_Br_2_ and a yet not identified Pb-S-Cl phase) in the form of NCs^25^. More recently, we found that Pb_4_S_3_Br_2_ can match epitaxially the CsPbX_3_ perovskite to form colloidal heterostructures, thus demonstrating a remarkable synthetic and structural compatibility^26^. Here we focus our attention on the lead sulfochlorides, demonstrating that the yet unknown Pb-S-Cl phase obtained through direct synthesis is Pb_3_S_2_Cl_2_, whose structure, here identified as monoclinic, is a distorted version of the cubic Pb_3_Se_2_Br_2_ prototype (Fig. 1, left reaction route)^31^. Conversely, the introduction of CsPbCl_3_ nanoclusters in the reaction medium, under comparable reaction conditions, produces Pb_4_S_3_Cl_2_/CsPbCl_3_ heterostructures while suppressing the formation of Pb_3_S_2_Cl_2_. Once the heterostructures are formed, the perovskite domain can be etched by exploiting the solubility of CsPbCl_3_ in polar solvents, while leaving the Pb_4_S_3_Cl_2_ domains intact. Hence, the use of CsPbCl_3_ as a disposable template yields colloidally stable Pb_4_S_3_Cl_2_ NCs that could not be obtained by direct synthesis due to the competitive nucleation of Pb_3_S_2_Cl_2_ (Fig. 1, top-right reaction route).

The reason why CsPbCl_3_ induces such phase selectivity lies in the fact that the structures of Pb_3_S_2_Cl_2_ and Pb_4_S_3_Cl_2_ are remarkably different^25,30^, to the point that only Pb_4_S_3_Cl_2_ can match epitaxially with the CsPbCl_3_ perovskite. Indeed, Pb_4_S_3_Cl_2_ meets two strict structural constraints: i) the presence of a perovskite-like atomic plane^26^, and ii) the continuity of the cationic subnetwork across the chalcohalide/perovskite interface^32^. Such conditions are not met for Pb_3_S_2_Cl_2_, whose homogeneous nucleation is, therefore, less favorable than the heterogeneous nucleation of Pb_4_S_3_Cl_2_ templated by the CsPbCl_3_ perovskite.

Overall, our method effectively combines the concept of sacrificial substrates, such as the alkali halide single crystals used for the large-area synthesis of films and ultrathin materials^33–36^, with the phase-selection capabilities ensured by the epitaxial templating, all in the liquid phase.

## Results

### Structure solution of Pb_3_S_2_Cl_2_ NCs

**Fig. 2:**
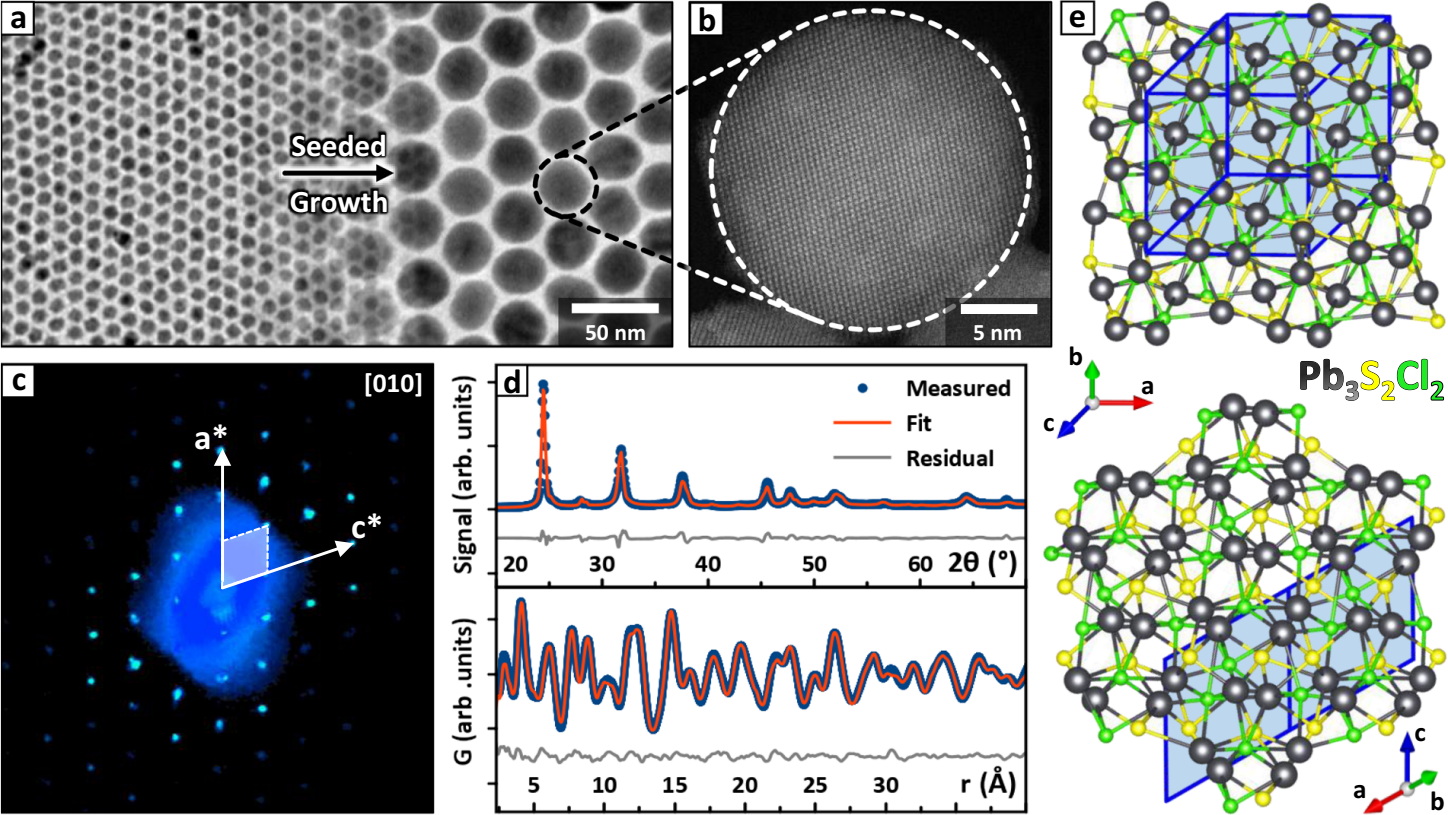
Pb_3_S_2_Cl_2_ NCs structure solution. **a** Pb_3_S_2_Cl_2_ NCs as synthesized (left) and after the accretion process (right). **b** HAADF-STEM image of a Pb_3_S_2_Cl_2_ NC. **c** [010] projection of the Pb_3_S_2_Cl_2_ reciprocal lattice measured by 3D-ED, together with a representation of the reciprocal lattice axes and unit cell. **d** XRPD (top) and PDF (bottom) fits obtained by refining the Pb_3_S_2_Cl_2_ structure in the *C*c space group (experimental data = blue, fit curves = orange, residuals = gray). The XRPD profiles are plotted in the Cu-K_α_ 2*θ* scale to ease the comparison with lab-grade diffraction patterns. **e** Two views of the *C*c monoclinic Pb_3_S_2_Cl_2_ structure are shown along the high symmetry directions of the corresponding pseudocubic cell ([100] top, [111] bottom). A projection of the monoclinic cell is overlaid in blue, and the directions of the corresponding lattice vectors are indicated by labeled arrows. Atoms color code: Pb = gray; S = yellow; Cl = green. Source data for **d** are provided as a Source Data file.

We start by discussing the synthesis and structure solution of lead sulfochloride NCs prepared by homogenous nucleation, that is, in the absence of halide perovskites. We recently reported the synthesis of the same NCs by a different procedure^25^, but in our previous work the small size of the particles and the heavy PbS contamination prevented us from determining the composition and crystal structure, which had therefore remained unknown to date. To solve the structure in the present work, we prepared large-size NCs via a two-step procedure, consisting of nucleation and seeded growth (Fig. 2a, b, see Supplementary Discussion, section 1 for details).

The synthesis yielded particles with a diameter of 29.5 ± 2.0 nm, as estimated by Transmission Electron Microscopy (TEM). Beyond this size the NCs became insoluble in the reaction medium, and the accretion process halted. The composition measured by SEM-EDX was Pb:S:Cl = 3.2:1.8:2.0, which is compatible with our previously advanced hypothesis of a Pb_3_S_2_Cl_2_ compound^25^. However, the X-Ray Powder Diffraction (XRPD) pattern could not be fitted based on the supposedly related Pb_3_Se_2_Br_2_ cubic prototype^25,31^, suggesting a different structure. Therefore, we exploited a combination of techniques to solve it. First, we performed a single-NC 3D Electron Diffraction (3D-ED)^37^ experiment to identify the unit cell parameters and a list of possible space groups (Fig. 2c). For some of them we also produced ab-initio solutions; however, a definitive structural model could not be obtained due to the small size of the NCs, which made the integrated intensity of the diffraction spots inaccurate. Thus, we exploited the information from 3D-ED to repeat the ab-initio structure solution from XRPD data with the software EXPO2014^38^, eventually obtaining a model close to the cubic Pb_3_Se_2_Br_2_ in terms of connectivity, but with a lower symmetry. Finally, we refined this model in both reciprocal and direct spaces on data collected at the Brookhaven National Laboratory synchrotron, on setups optimized for XRPD and Pair Distribution Function (PDF) experiments respectively (Fig. 2d).

The solution was found in the monoclinic *C*c space group (Fig. 2e, see Supplementary Discussion, section 2 for details), capturing deviations from the Pb_3_Se_2_Br_2_ cubic prototype that are likely due to the small ionic radii of S^2−^ and Cl^−^. Density Functional Theory calculations (DFT, see Supplementary Discussion, section 3) confirmed that as a bulk crystal the monoclinic Pb_3_S_2_Cl_2_ would be more stable than a hypothetical cubic polymorph by 164 kcal/mol. The ionic radii are also likely to play a major role in favoring the Pb_3_S_2_Cl_2_ phase over Pb_4_S_3_Cl_2_ for free-standing lead sulfochloride NCs. Indeed, Pb_3_E_2_X_2_ appears to be favored if ${r}_{{E}^{2-}}/{r}_{{X}^{-}}\approx 1$ (Se^2−^/Br^−^ = 1.01; S^2−^/Cl^−^ = 1.02)^31^, while ${r}_{{E}^{2-}}/{r}_{{X}^{-}} < 1$ favors Pb_4_E_3_X_2_ (S^2−^/Br^−^ = 0.94; S^2−^/I^−^ = 0.84)^25,30,39^. Indeed, in both structures Pb^2+^ is surrounded by eight anions, and S^2−^ features a distorted octahedral coordination. Conversely, the coordination of the halide changes: in Pb_3_S_2_Cl_2_ the smaller Cl^−^ ions share the same octahedral coordination as S^2−^, while the larger Br^−^ and I^−^ ions in Pb_4_S_3_X_2_ are surrounded by seven Pb^2+^ ions in a pentagonal bipyramidal configuration. The reason is likely that bulkier anions can accommodate an enlarged coordination environment. Interestingly, Pb_3_S_2_Cl_2_ is the only lead chalcohalide where Cl^−^ is coordinated by an octahedron of Pb^2+^ ions. Such coordination has been recently proposed on the surface of PbS NCs synthesized in excess of PbCl_2_ to account for the formation of a Cl-rich shell that improves the optical properties^40–43^, suggesting that these NCs might be passivated by a layer of some lead sulfochloride compound. For further comparisons between the structures of Pb_3_S_2_Cl_2_ and Pb_3_S_2_X_2_ compounds see Supplementary Discussion, section 4, which includes the structure refinements of Pb_4_S_3_Br_2_ and Pb_4_S_3_I_2_ NCs and a comparison of PDF profiles by RootProf^44^.

### Compatibility of lead sulfochlorides with CsPbX_3_

**Fig. 3:**
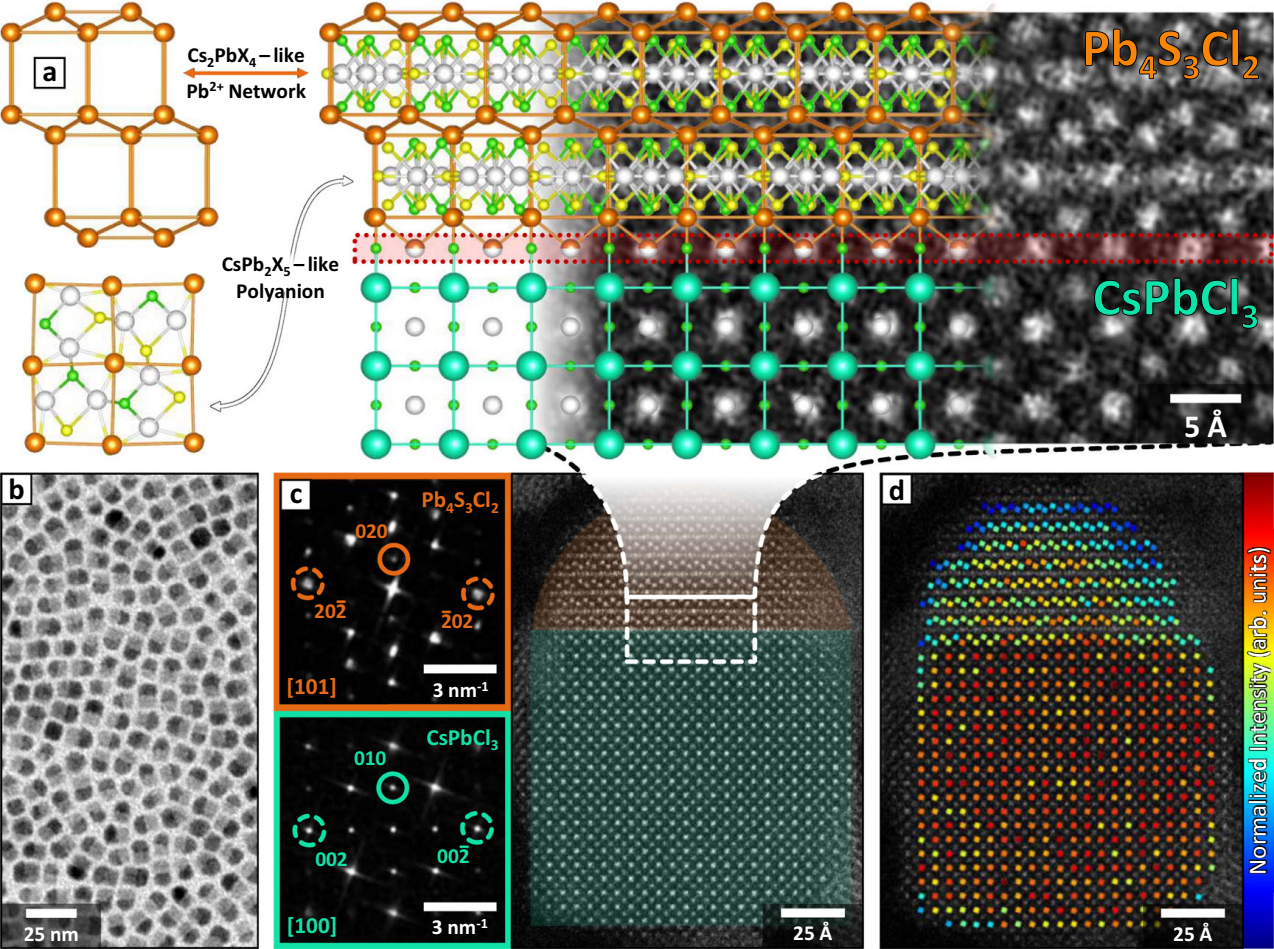
Pb_4_S_3_Cl_2_/CsPbCl_3_ epitaxial heterostructures. **a** On the left, models of the Cs_2_PbX_4_-like Pb^2+^ subnetwork (orange Pb^2+^) and of the CsPb_2_X_5_-like [Pb_2_S_3_Cl_2_]^−^ polyanion (white Pb^2+^, yellow S^2-^, green Cl^−^) found in Pb_4_S_3_Cl_2_. On the right, model of the Pb_4_S_3_Cl_2_/CsPbCl_3_ epitaxial interface superimposed on a close-up view of **c**, highlighting the continuity of the Pb^2+^/Cs^+^ cationic subnetwork. The Pb_4_S_3_Cl_2_ structure has been represented by adapting the Pb_4_S_3_Br_2_ structure. Atoms color code: Cs = cyan; Pb = orange/white; S = yellow; Cl = green. **b** Low resolution TEM image of as-synthesized Pb_4_S_3_Cl_2_/CsPbCl_3_ heterostructures. **c** HAADF-STEM image of a single Pb_4_S_3_Cl_2_/CsPbCl_3_ heterostructure. Insets: FFTs of the Pb_4_S_3_Cl_2_ (top) and CsPbCl_3_ (bottom) domains. The spots circled in a solid line correspond to planes parallel to the heterostructure interface and to each other; those circled in a dashed line are instead perpendicular to the interface and share similar periodicities, ensuring the match of the two lattices. **d** Column intensity map of the Pb-containing columns in the perovskite phase and of the Pb columns in the Cs_2_PbX_4_-like subnetwork of the Pb_4_S_3_Cl_2_ domain. The color in the intensity map correlates with the total intensity scattered from the corresponding atomic column (red = higher intensity; blue = lower intensity). The map is compatible with the interface model depicted in Fig. 3a, and highlights the preservation and merging of the cationic subnetwork of the two phases across the interface. Source data for **d** are provided as a Source Data file.

Our prior studies demonstrated that the lead sulfobromide Pb_4_S_3_Br_2_ can grow epitaxially on lead halide perovskite domains^26^, suggesting that other isostructural compounds, i.e., Pb_4_S_3_I_2_ and a hypothetical Pb_4_S_3_Cl_2,_ would do the same. Conversely, the Pb_3_S_2_Cl_2_ structure we hereby solved is remarkably different from that of the Pb_4_S_3_X_2_ compounds, to the point that the epitaxial compatibility with CsPbX_3_ would be lost. This prompted us to think that the synthesis of a sulfochloride/perovskite heterostructure would produce domains of the yet unknown compound Pb_4_S_3_Cl_2_ instead. Indeed, the Pb_4_S_3_X_2_/CsPbX_3_ match, regardless of the halide, is strictly structure specific. First, both the chalcohalide and the perovskite share a plane of Pb^2+^ ions arranged in a square grid, which serves as an interface between the two domains (in red in Fig. 3a, right)^26^. No such plane can be found in Pb_3_S_2_Cl_2_. Moreover, Pb_4_S_3_X_2_ shares deep similarities with some of the Cs-Pb-X phases and the heterostructures they form with CsPbX_3_, which are easily overlooked at first sight. Indeed, the Cs-Pb-X compounds share a common cationic subnetwork of Cs^+^ ions, which in Cs-Pb-X/Cs-Pb-X heterostructures is preserved across the interface and ensures the lattice compatibility^32^. Remarkably, similar connectivity is found across the Pb_4_S_3_X_2_/CsPbX_3_ interface. Relevant examples for our discussion are the CsPb_2_X_5_/CsPbX_3_ heterostructures^45,46^. In CsPb_2_X_5_, the Cs^+^ subnetwork encloses layers of [Pb_2_X_5_]^−^ bidimensional polyanions^32^. Remarkably, Pb_4_S_3_X_2_ is almost isostructural to CsPb_2_X_5_, being formed by a Pb^2+^ subnetwork enclosing [Pb_2_S_3_X_2_]^−^ polyanions of identical geometry. The only difference is the presence of an extra layer of Pb^2+^ ions in between each polyanion, that is needed to maintain the charge balance (Fig. 3a, left). This makes the Pb^2+^ subnetwork of Pb_4_S_3_X_2_ resemble that of another Cs-Pb-X phase, namely Cs_2_PbX_4_. Consequently, the cationic subnetwork of Pb_4_S_3_X_2_ is similar to that found in Cs-Pb-X compounds and can find its natural prosecution in the CsPbX_3_ perovskite (Fig. 3a, right), ensuring the stability of the interface and remarking the pivotal role of ionic subnetworks in the formation of colloidal heterostructures. Deeper insights into the heterostructure interface connectivity and the similarities with Cs-Pb-X/Cs-Pb-X heterostructures can be found in the Supplementary Discussion, section 5.

### Synthesis of Pb_4_S_3_Cl_2_/CsPbCl_3_ heterostructures

To test whether the natural affinity of the Pb_4_S_3_X_2_ phases for lead halide perovskites would lead to the formation of Pb_4_S_3_Cl_2_, we adapted our previously reported method to attempt the synthesis of Pb_4_S_3_Cl_2_/CsPbCl_3_ heterostructures^26^. In the first step of the procedure, CsPbCl_3_ nanoclusters, prepared separately, were reacted with elemental sulfur in the presence of lead oleate and dodecanethiol at 200 °C, indeed producing heterostructures. The resulting particles were recovered through antisolvent precipitation, and then treated with a solution of PbCl_2_, oleylamine, and oleic acid to restore their colloidal stability (see Supplementary Discussion, section 6). The heterostructures were homogeneous in terms of size and shape (Fig. 3b), and no single-phase perovskite or sulfochloride NCs were observed.

High-Angle Annular Dark-Field Scanning TEM (HAADF-STEM) showed that the heterostructures were composed of two highly crystalline domains jointed along a flat interface spanning across the entire nanoparticle (Fig. 3a, c). As expected, the fast Fourier transform (FFT) of the HAADF-STEM image revealed that the CsPbCl_3_ domain matches with the perovskite structure, while that of the lead sulfochloride domain is incompatible with Pb_3_S_2_Cl_2_. Instead, the unit cell parameters and the overall FFT symmetry match with what is expected for a Pb_4_S_3_X_2_ domain based on our previous observations on Pb_4_S_3_Br_2_/CsPbX_3_ heterostructures (insets of Fig. 3c)^26^. Such phase selectivity was granted by the transformation of CsPbCl_3_ nanoclusters into NCs during the early stages of the reaction^26^. The perovskite NCs then acted as phase-selective heterogeneous nucleation seeds for Pb_4_S_3_Cl_2_, providing a significant advantage over the homogeneous nucleation of Pb_3_S_2_Cl_2_, which was indeed suppressed.

The predicted structure of the Pb_4_S_3_Cl_2_/CsPbCl_3_ interface, superimposed in Fig. 3a to a high-resolution HAADF-STEM image of the heterostructure, was further confirmed by highlighting the Pb-containing atomic columns involved in the Pb^2+^ cationic subnetwork through the quantitative analysis of HAADF-STEM images (Fig. 3d). Indeed, it is worth noting that a prosecution of the chalcohalide-Pb^2+^ subnetwork, identified in Fig. 3a with the perovskite-Cs^+^ subnetwork in analogy with the CsPb_2_X_5_/CsPbX_3_ heterostructures, can also be found in the Pb^2+^ subnetwork of the perovskite domain, as it shares the same symmetry as the Cs^+^ one (see Supplementary Discussion, section 5 for details).

### Selective etching of the CsPbCl_3_ domain

**Fig. 4:**
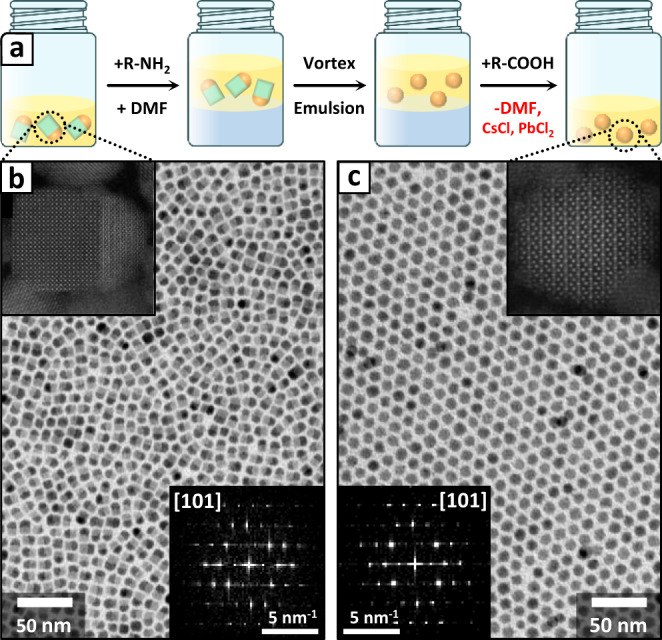
Selective etching of Pb_4_S_3_Cl_2_/CsPbCl_3_ heterostructures. **a** Scheme of the DMF-assisted etching procedure developed to transform the Pb_4_S_3_Cl_2_/CsPbCl_3_ heterostructures into Pb_4_S_3_Cl_2_ NCs in the presence of surfactants (R-NH_2_ = oleylamine, R-COOH = oleic acid). The cyan cubes and the orange spheres represent the CsPbCl_3_ and the Pb_4_S_3_Cl_2_ domains of the heterostructures, respectively. **b** TEM image of the starting heterostructures. The top inset shows an HAADF-STEM image of one heterostructure prior to the etching, while the bottom inset shows the FFT of the chalcohalide domain of the same image. **c** TEM image of the etched NCs. The top inset shows an HAADF-STEM image of one NC after the etching, while the bottom inset shows the FFT of the same image. The two FFTs demonstrate that the Pb_4_S_3_Cl_2_ domains retain the same crystal structure before and after the etching procedure.

Our templated synthesis approach could be pushed a step further as we took advantage of the solubility of CsPbCl_3_ in polar solvents to selectively etch the perovskite domains and recover phase-pure Pb_4_S_3_Cl_2_ NCs (Fig. 4). Briefly, the heterostructures dispersed in hexane were first treated with oleylamine to improve the colloidal stability of the etched NCs in nonpolar media. Then, we added to the dispersion an equal volume of dimethylformamide (DMF), and the resulting mixture was vortexed for 30 s, forming an emulsion. After its complete separation, DMF was removed, and predried oleic acid was introduced in the NCs hexane dispersion. Subsequently, the particles were recovered through antisolvent precipitation followed by a centrifugation, and redispersed in toluene for further use. The procedure yielded remarkably uniform Pb_4_S_3_Cl_2_ NCs, whose structure was confirmed via high-resolution HAADF-STEM imaging and analysis of the lattice by means of FFTs. Interestingly, the NCs appeared to be sphere-shaped despite the original chalcohalide domains in the heterostructures being hemispherical. We attribute this change of morphology to the need of lowering the surface energy of the extended and non-passivated facet left after the dissolution of the perovskite. It is likely that the excess ions released by the etching process will recrystallize on the chalcohalide domains, thus reshaping them into a more stable spherical morphology. See Supplementary Discussion, section 7 for details on the etching of heterostructures.

### Optoelectronic properties of lead sulfochloride NCs

**Fig. 5:**
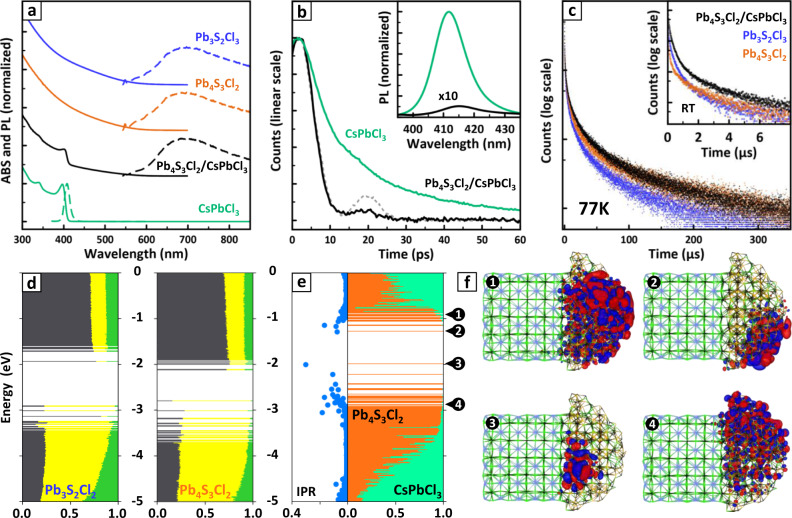
Optoelectronic properties of lead sulfohalide NCs and Pb_4_S_3_Cl_2_/CsPbCl_3_ heterostructures. **a** Absorption (ABS, solid lines) and photoluminescence (PL, dashed lines) spectra of free-standing Pb_3_S_2_Cl_2_, Pb_4_S_3_Cl_2_, CsPbCl_3_ NCs and of Pb_4_S_3_Cl_2_/CsPbCl_3_ heterostructures. The CsPbCl_3_ NCs were prepared for comparison purposes by heating the CsPbCl_3_ precursor nanoclusters (see Supplementary Discussion, section 6). **b** Decay curves of the ∼410 nm PL from CsPbCl_3_ as free-standing NCs and in the Pb_4_S_3_Cl_2_/CsPbCl_3_ heterostructures. The dashed gray line is the time response of the experimental setup. Inset: PL spectra of CsPbCl_3_ as free-standing NCs and in the heterostructures, showing a drastic quenching in the latter case. The PL intensity of the heterostructures is multiplied ×10 to make it more visible. The slight redshift of the heterostructure PL maximum is likely due to the relaxation of quantum confinement of the perovskite domain caused by the partial delocalization of the exciton in the chalcohalide domain. **c** Decay curves of the ∼1.8 eV (∼690 nm) emission of the three samples at room temperature and at 77 K (inset). Color code for **a**–**c**: cyan = CsPbCl_3_, blue = Pb_4_S_3_Cl_2_, orange = Pb_3_S_2_Cl_2_, black = Pb_4_S_3_Cl_2_/CsPbCl_3_ heterostructures. **d** Electronic structure of free-standing Pb_3_S_2_Cl_2_ (left) and Pb_4_S_3_Cl_2_ (right) NC models computed at the DFT/PBE level of theory. The color code indicates the elemental contribution to each molecular orbital (Pb = gray; S = yellow; Cl = green). **e** Electronic structure of the Pb_4_S_3_Cl_2_/CsPbCl_3_ heterostructure model computed at the DFT/PBE level of theory, color coded by domain (Pb_4_S_3_Cl_2_ = orange; CsPbCl_3_ = cyan). The Inverse Participation Ratio (IPR, left panel) quantifies the degree of localization of a state (1/*N*_atoms_ = completely delocalized; 1 = localized on one single atom), and indicates that most of the states surrounding the band gap are localized trap states. **f** Representation of molecular orbitals corresponding to band edge delocalized states (1,4) and to band edge trap states (2,3). In both cases, the band edge states are localized on the Pb_4_S_3_Cl_2_ domain. Source data for **a**–**e** are provided as a Source Data file.

Obtaining both Pb_3_S_2_Cl_2_ and Pb_4_S_3_Cl_2_ NCs, and the latter both in the form of free-standing NCs and heterostructures with CsPbCl_3_, gave us the opportunity to investigate the impact of stoichiometry, structure, and presence of a heterojunction on the optoelectronic properties of lead sulfochlorides. Figure 5 summarizes the experimental results, along with data from DFT calculations. The free-standing Pb_3_S_2_Cl_2_ and Pb_4_S_3_Cl_2_ NCs exhibited basically overlapping and featureless absorption spectra, with onset at ∼1.8 eV (∼690 nm, Fig. 5a, solid blue and orange plots). The Tauc plot analysis suggested an indirect band gap of ∼1.8 eV in both cases, in agreement with the DFT predictions (see Supplementary Discussion, sections 3 and 8). This is in stark contrast with the direct bandgap observed for lead chalcogenides (PbE) and many lead halides (e.g., CsPbX_3_), remarking that the electronic properties of lead chalcohalides are not straightforwardly related to those of chemically similar compounds. A comparable absorption profile was found in the heterostructures spectrum, combined with the blunt absorption edge of the direct-gap CsPbCl_3_ domain (∼3.1 eV = 400 nm^24,47^, Fig. 5a, solid black plot). Such edge was instead sharp for free-standing CsPbCl_3_ NCs (Fig. 5a, solid cyan plot), suggesting an intimate electronic connection between the two domains in the heterostructures. This was further supported by the almost complete suppression of the CsPbCl_3_ photoluminescence (PL) when the perovskite domain was involved in the heterojunction (Fig. 5b, inset).

DFT calculations suggested that the cause for the PL quenching was the nearly type-I band alignment between Pb_4_S_3_Cl_2_ and CsPbCl_3_ (Fig. 5e, see also Supplementary Discussion, section 3). Interestingly, this contrasts with the structurally equivalent Pb_4_S_3_Br_2_/CsPbBr_3_ heterostructures investigated in our previous work, which were instead identified as quasi-type-II junctions^26^. Indeed, the molecular orbitals corresponding to the band edge states of the heterostructures are strongly localized on the sulfochloride domain (Fig. 5f). It is worth noting that the predicted type-I alignment should promote the migration of photocarriers into the sulfochloride domain of the heterostructure. Indeed, the decay of the barely detectable PL of CsPbCl_3_ in the heterostructures was strongly accelerated if compared with that of free-standing CsPbCl_3_ NCs (*τ*_avg_ ~8 ps vs ~18 ps, Fig. 5b).

Owing to the relaxation of the momentum conservation at the nanoscale, both the free-standing sulfochlorides and the heterostructures exhibited a broad PL peak at ∼1.8 eV (∼690 nm, see Fig. 5a, dashed lines), indicating in all the cases a sulfochloride band edge emission. Such emission was negligible at room temperature (PLQY < 1%) due to the efficient thermal quenching. However, lowering the temperature to 77 K resulted in a ~100-fold intensification of the PL, accompanied in the case of heterostructures by a brightening of the residual, yet still negligible, CsPbCl_3_ emission. In all three cases, the PL kinetics of the lead chalcohalides was markedly non-exponential, with a dominant sub-microsecond drop followed by a slower decay (Fig. 5c). The process was slightly slower in the heterostructures with respect to both free-standing Pb_3_S_2_Cl_2_ and Pb_4_S_3_Cl_2_ NCs, suggesting that the CsPbCl_3_ domain might suppress some trapping losses by passivating part of the sulfochloride domain surface. This is also consistent with our observation of a more intense PL. At low temperatures, all the PL decays became substantially longer. Fitting the dataset with an Arrhenius function yielded for all the samples an activation energy of ∼15 meV. Remarkably, the spectral shape of all samples followed a nearly identical trend with the temperature, showing a progressive shift to ∼1.5 eV (∼827 nm) and a band narrowing, further corroborating the strong similarities between the optoelectronic properties of these three systems. See Supplementary Discussion, section 8 for details on the spectroscopic characterizations.

In general, our analysis indicates that the properties of lead sulfochlorides are largely independent of both stoichiometry and structure. This completes our preliminary observations on Pb_4_S_3_Br_2_ and Pb_4_S_3_I_2_ NCs, that shared nearly identical absorption spectra despite containing different halides^25^. We rationalized such behavior as a consequence of two factors. First, in these materials the band edge states feature a prominent participation of Pb^2+^ and S^2−^, while the halides mostly contribute to states buried deep in the valence band, making the electronic properties almost halide-insensitive. Second, regardless of the structure of the specific chalcohalide, the geometry and connectivity of the coordination polyhedra surrounding both Pb^2+^ and S^2−^ is the same, making the electronic properties basically structure-insensitive.

## Discussion

In this work, we have exploited the epitaxial templating effect of CsPbCl_3_ to control the synthesis of lead sulfohalide NCs through the formation of Pb_4_S_3_Cl_2_/CsPbCl_3_ heterostructures. Indeed, while a direct synthesis approach (i.e., in the absence of CsPbCl_3_ acting as a template) yielded Pb_3_S_2_Cl_2_ NCs, only the selective etching of the heterostructures perovskite domain allowed us to recover free-standing Pb_4_S_3_Cl_2_ NCs. This ultimately means that we could select the reaction product between two competing phases, a problem of great relevance in the synthesis of colloidal inorganic nanomaterials. Both the obtained lead sulfochlorides shared remarkably similar optoelectronic properties. This, combined with our previous observation on bromine- and iodine-based sulfohalides^25^, suggests that lead sulfohalide compounds are mostly stoichiometry- and structure-insensitive. Interestingly, the discovery of the Pb_3_S_2_Cl_2_ structure demonstrates that Cl^−^ in lead chalcohalides can be coordinated by an octahedron of Pb^2+^ ions, differently from Br^−^ and I^−^. This supports the recent observations of a PbCl_x_-rich shell on the surface of PbS NCs synthesized using PbCl_2_ as a precursor, a condition that is remarkably similar to our synthetic protocol and might indeed lead to the formation of a lead sulfochloride surface layer^40–43^.

We remark that our use of perovskite NCs as disposable and phase-selective epitaxial templates parallels that of reaction-directing groups in traditional organic chemistry and catalysis^27^, and allowed us to design the phase-selective synthesis of two yet unknown materials (Pb_3_S_2_Cl_2_ and Pb_4_S_3_Cl_2_) based on structural considerations only. Such an approach to a deterministic synthesis of NCs may be extended to other phases with known or predictable epitaxial relations, taking advantage of the vast library of already reported nanomaterials as starting templates. For example, in the field of chalcohalides it might lead to yet unknown selenium- and tellurium-based materials, or even to compounds where the halide is replaced by pseudohalide ions (e.g., CN^−^ and SCN^−^). In conclusion, we envision that our phase-selective templating approach will open new routes for the colloidal syntheses of nanomaterials which are now hindered by an excessive activation energy for the homogeneous nucleation, or by the competitive formation of undesired phases.

## Methods

### Chemicals

1-octadecene (ODE, technical grade, 90%), oleic acid (OA, technical grade, 90%), oleylamine (OLA, technical grade, 70%), lead(II) chloride (PbCl_2_, 98%), lead(II) bromide (PbBr_2_, 98%), lead(II) iodide (PbI_2_, 98%), lead(II) thiocyanate (Pb(SCN)_2_, 99.5%), lead(II) acetate trihydrate (Pb(OAc)_2_·3H_2_O, 99.99%), cesium(I) carbonate (Cs_2_CO_3_, 99%), dodecanethiol (DDT, 99.9%), and sulfur powder (S, 99.99%) were purchased from Sigma‐Aldrich. All reagents were used as received without any further purification.

### Synthesis: S-ODE stock solution for Pb_3_S_2_Cl_2_ NCs

0.064 g (2 mmol) of S powder were mixed with 10 mL of ODE (pre-degassed at 120 °C under vacuum for an hour) in a 20 mL glass vial inside a glove box filled with N_2_. The resulting mixture was sonicated until the complete dissolution of the sulfur powder.

### Synthesis: Pb_3_S_2_Cl_2_ nanocrystals (NCs)

0.21 g of PbCl_2_ (0.8 mmol) were solubilized at 120 °C in a mixture of 2 mL of OLA, 2 mL of OA and 20 mL of ODE in a 100 mL flask. The solution was then heated up to 170 °C, and 2 mL of the S-ODE stock solution, pre-heated at 150 °C, were swiftly injected. The NCs formed ∼10–30 s after the injection, as revealed by the color change of the solution from pale yellow to red. After 1 min, the reaction was quenched by cooling the flask with a room-temperature water bath. The NCs were recovered by adding 40 mL of ethyl acetate, followed by a centrifugation at 6000 rpm (∼2940 × *g*) for 5 min. The precipitate was discarded, and the supernatant was suspended in hexane or toluene. If needed, the sample could be washed multiple times by adding ethyl acetate and repeating the centrifugation. The maximum diameter for Pb_3_S_2_Cl_2_ NCs obtained by direct synthesis was 7–8 nm (see Supplementary Discussion, section 1).

### Synthesis: accretion of larger Pb_3_S_2_Cl_2_ NCs

Larger NCs were obtained through a seeded growth approach, performed by using as starting seeds Pb_3_S_2_Cl_2_ NCs prepared as detailed above. A stock solution of reagents was prepared by dissolving 0.2966 g of PbCl_2_ (1.06 mmol) and 0.6898 g of Pb(SCN)_2_ (2.13 mmol) in a mixture of 4 mL of OLA, 4 mL of OA, and 40 mL of ODE in a 100 mL flask at 120 °C. During its preparation, the stock solution must not exceed this temperature to avoid the thermal decomposition of the thiocyanate, which would cause the nucleation of PbS NCs^25^. Once the two solids were completely solubilized, the stock solution was cooled to room temperature, filtered with a 0.2 μm PTFE syringe filter, and loaded on a syringe pump. Then, a crude Pb_3_S_2_Cl_2_ reaction mixture in ODE was obtained by following the procedure detailed in the previous paragraph up to before the addition of ethyl acetate. 12 ml of such solution were heated to 170 °C, and the stock solution of PbCl_2_ and Pb(SCN)_2_ was added dropwise at a controlled rate of 10 mL/h with the help of a syringe pump. Finally, the reaction mixture was washed with ethyl acetate as described in the previous paragraph, and the NCs were suspended in hexane. A last step of filtration performed with a 0.2 μm PTFE syringe filter allowed to remove any impurity of PbS that may have formed during the accretion process. The so-obtained Pb_3_S_2_Cl_2_ NCs measured 29.5 ± 2.0 nm on average, and were used to solve the structure of the material by a combination of 3D-ED, XRPD, and PDF experiments. See Supplementary Discussion, section 1 for additional details on the accretion process.

### Synthesis: Pb_4_S_3_Br_2_ (or Pb_4_S_3_I_2_) NCs

NCs of Pb_4_S_3_Br_2_ (and Pb_4_S_3_I_2_) were prepared following our previously published method^25^. In short, 0.2 mmol of PbBr_2_ (or PbI_2_) and 0.2 mmol of Pb(SCN)_2_ where dissolved in a mixture of 10 mL ODE and 250 μL of OLA and OA at 120 °C in a 25 mL three-necked flask. Then, the solution was quickly heated (∼20 °C/min) and started turning from light-yellow to bloody red above 150 °C while the NCs nucleated and grew. The reaction was quenched by cooling the flask in a water bath. The NCs were recovered by simple centrifugation or by ethyl-acetate assisted precipitation followed by centrifugation (6000 rpm for 5 min in both cases, ∼2940 × *g*). The so-obtained Pb_4_S_3_Br_2_ and Pb_4_S_3_I_2_ NCs were used to refine the structure of both materials by XRPD and PDF experiments (see Supplementary Discussion, section 4).

### Synthesis: Cs-oleate precursor for CsPbCl_3_ nanoclusters

The Cs-oleate precursor was prepared by increasing the concentration of oleic acid in comparison to our previously reported method^26^. In a typical synthesis, Cs_2_CO_3_ (0.652 g, 2 mmol), OA (5 mL, 15 mmol) and ODE (17.5 mL) were loaded into a 50 mL 3-neck flask, dried for 1 h at 110 °C and then heated under N_2_ to 150 °C until the solution turned clear. The resulting mixture was transferred into a N_2_ filled glass vial, which was stored inside a glovebox for further use.

### Synthesis: PbCl_2_ stock solution for heterostructures

PbCl_2_ powder (0.556 g, 2 mmol), OA (5 mL), and OLA (5 mL) were mixed with ODE (30 mL) in a 100 mL three neck round bottom flask. The reaction mixture was dried/degassed under vacuum for 15 min at 110 °C. Then, the flask was filled with N_2_ and the temperature was raised to 150 °C. After complete dissolution of PbCl_2_ salt, the solution was cooled down to room temperature (25 °C) and transferred into a N_2_-filled glass vial.

### Synthesis: Pb(OA)_2_ stock solution for heterostructures

The lead oleate stock solution was prepared following our previously reported method^26^. Briefly, Pb(OAc)_2_·3H_2_O powder (0.76 g, 2 mmol) and OA (1.3 mL), were mixed with ODE (18.7 mL) in a 50 ml three neck round bottom flask. The reaction mixture was degassed under vacuum for 1 h at 110 °C and then heated under N_2_ to 150 °C until all Pb(OAc)_2_·3H_2_O reacted with OA. Thereafter, the solution was cooled down to room temperature (25 °C) and transferred into N_2_-filled glass vials.

### Synthesis: CsPbCl_3_ nanoclusters

CsPbCl_3_ nanoclusters were synthesized following a previously reported method^26^. Briefly, 4 mL of the above-mentioned PbCl_2_ stock solution were transferred into the N_2_ filled 20 ml glass vial. Thereafter, 0.25 mL of the Cs-oleate precursor were injected into the PbCl_2_ stock solution at 40 °C and the resulting mixture was kept under stirring for 20 min. After about 20 min, the mixture (turbid white) was centrifuged at 8000 rpm (∼3935 × *g*) for 5 min, the supernatant was discarded, and the precipitate was redispersed in 0.9 mL of degassed ODE.

### Synthesis: Pb_4_S_3_Cl_2_/CsPbCl_3_ heterostructures

4.0 mL of degassed ODE were added to a 20 mL glass vial under N_2_. The vial was heated to 200 °C, and 100 μL of the above-mentioned Pb(OA)_2_ solution and 20 μL DDT (diluted in 200 μL predried ODE) were added into the reaction system. Then, the mixture of S (0.1 mL) and CsPbCl_3_ nanoclusters (0.9 mL) was rapidly injected into the reaction mixture. The reaction was allowed to proceed for 5 min and was subsequently quenched by immersing the vial in an ice and water bath. The crude solution was then centrifuged by adding methyl acetate (with volume ratio of 1 to 1) and the precipitate was redispersed in toluene. See Supplementary Discussion, section 6 for details.

### Synthesis: surface treatment of heterostructures

A PbCl_2_ solution, separately prepared by dissolving PbCl_2_ salt (1 mmol) in ODE (15 mL) in the presence of oleic acid (2.5 mL) and oleylamine (2.5 mL), was added to the toluene dispersion containing the heterostructures, and the mixture was vortexed for 1 min. Then, the solution was centrifuged by adding methyl acetate and the precipitate was redispersed in hexane or toluene for further use. The role of this treatment is to reconstruct the surface of the CsPbCl_3_ perovskite domains after the antisolvent-assisted precipitation, and to ensure the long-term colloidal stability of the sample.

### Synthesis: CsPbCl_3_ NCs

4.0 mL of ODE were added to a 20 mL glass vial under air and heated to 150 °C, then 1.0 mL of the above-mentioned seed cluster solution (CsPbCl_3_, without S-ODE) was swiftly injected. The mixture was annealed for 5 min and subsequently cooled down by using an ice water bath. The resulting mixture was centrifuged at 8000 rpm (∼3935 × *g*) for 5 min, the supernatant was discarded, and the precipitate was dispersed in 4 mL of toluene.

### Synthesis: etching of Pb_4_S_3_Cl_2_/CsPbCl_3_ heterostructures

1 mL of heterostructures dispersed in hexane (corresponding to one entire batch of heterostructures) was treated first with oleylamine (60 μL). Then, an equal volume ratio of dimethylformamide (DMF, 1 mL) was added to the heterostructures dispersion and the resulting mixture was vortexed for 30 s. After the complete phase separation of solvents (2–3 min), DMF was removed by using a syringe and pre-dried oleic acid (60 μL) was introduced in the hexane dispersion. Subsequently, the now etched NCs were centrifuged by adding methyl acetate (with 1 to 1 volume ratio) and were redispersed in toluene for further use. See Supplementary Discussion, section 7 for details.

### Characterization: TEM

Bright-field TEM images of the samples were acquired with a JEOL-1100 transmission electron microscope operating at an acceleration voltage of 100 kV. The samples were prepared by drop casting diluted solutions of NCs or heterostructures onto carbon film-coated 200 mesh copper grids for low-resolution TEM.

### Characterization: Lab-grade XRPD

Lab-grade XRPD analyses were performed in *θ*:2*θ* scan mode on a Panalytical Empyrean diffractometer, equipped with a 1.8 kW Cu-K_α_ ceramic anode working at 45 kV–40 mA and a PIXcel3D detector. XRPD data were acquired on samples in the form of dry powders or drop-casted solutions; the measurements were carried out in air at room temperature using a zero-diffraction silicon substrate.

### Characterization: HAADF-STEM

High-resolution HAADF-STEM images were acquired with a probe-corrected cubed Thermo Fisher Scientific Themis Z Microscope operating at 300 kV with a probe semi-convergence angle of 20.5 mrad. For a quantitative analysis of the HAADF-STEM image, the intensities of the individual atomic columns in a single heterostructure were analyzed by using the StatSTEM software^48^. The color code in the intensity map correlates with the total intensity scattered from each atomic column. The intensity is calculated by fitting a Gaussian function to each atomic column: the intensity value of a column equals the volume of its Gaussian peak.

### Characterization: 3D-ED

3D-ED data were collected on a Zeiss Libra TEM operating at 120 kV and equipped with a LaB_6_ source. Data were acquired in STEM mode after defocusing the beam to achieve a parallel illumination of the sample. A beam size of about 150 nm in diameter was obtained by inserting a 5 μm C2 condenser aperture. A mild illumination was adopted to avoid any alteration or amorphization of the sample, and to slow down the accumulation of organic contaminants. 3D-ED data were recorded with an ASI Timepix detector^49^, which is able to detect single electrons and deliver a pattern that is virtually background-free. The camera length was 180 mm, with a theoretical resolution limit of 0.75 Å.

### Characterization: synchrotron XRPD and PDF data collection

Synchrotron diffraction data were collected at the 28ID-2 beamline of the National Synchrotron Light Source (NSLS-II) of the Brookhaven National Laboratory with an X-ray energy of 67.17 keV (0.1846 Å) and a 0.5 mm × 0.5 mm beam size. A Perkin Elmer XRD 1621 digital imaging detector (2048 × 2048 pixels and 200 × 200 µm pixel size) was mounted orthogonal to the beam path in two positions: 228 mm and 1365 mm downstream of the sample. Samples of Pb_3_S_2_Cl_2_, Pb_4_S_3_Br_2_, and Pb_4_S_3_I_2_ were measured in both setups, optimized for PDF and XRPD measurements, respectively. Lanthanum hexaboride (LaB_6_) was measured as a standard material to calibrate the wavelength and the detector geometry, including the sample-to-detector distance. An empty capillary was measured for background estimation. Diffraction images were azimuthally integrated and converted into intensity profiles vs 2*θ* and vs momentum transfer $Q=4{{\pi }}{{\sin }}\vartheta /{\lambda }_{X-{ray}}$ by using the FIT2D program^50^. PDF profiles were calculated up to interatomic distances *r* of 50 Å from the *Q* profiles with the program PDFGetX3^51^. The parameters for PDF calculation (background subtraction, scale factor, minimum and maximum values of *Q*, degree of data-correction polynomial) were optimized on individual PDF profiles, to avoid large termination effects and preserve the signal to noise ratio. The *Q*_max_ values were 23.7 Å^−1^ for Pb_3_S_2_Cl_2_ and 22.0 Å^−1^ for Pb_4_S_3_Br_2_ and Pb_4_S_3_I_2_.

### Characterization: NCs structure solution and refinement

The solution and refinement of the Pb_3_S_2_Cl_2_ NCs structure was performed through a combination of 3D-ED, XRPD, and PDF analyses. 3D-ED^37,52^ was used to determine the starting unit cell parameters and a set of possible space groups. In the process, the software ADT3D^53^ was used. XRPD was exploited to select the most promising space groups for the structure solution, extract the integrated intensities of reflections and perform the ab-initio structure solution by Direct Methods. In the process, the software EXPO2014^38^ was used. Both XRPD and DFT data were exploited to refine the structure of NCs, by alternating the refinement in the direct (PDF) and reciprocal (XRPD) spaces. To analyze the PDF profiles, the PDFGUI^44,54,55^ and DiffPy-CMI^16^ software packages were used. The same refinement strategy was applied to Pb_4_S_3_Br_2_ and Pb_4_S_3_I_2_ NCs, while the structure solution step was not needed because the starting structure was already known^25,30^. All the crystal structure illustrations shown in this work were produced by using the software VESTA^56^. Additional details are provided in the Supplementary Discussion, section 4.

### Characterization: optical spectroscopies

Absorbance and photoluminescence (PL) spectra of solution NCs and heterostructures were recorded on a Cary 500 spectrophotometer and Cary Eclipse spectrofluorometer, respectively. The spectra were measured in 10 × 10 mm quartz cuvettes. A wavelength of 350 nm was used for excitation during the collection of PL spectra. Regarding the extended spectroscopy: for PL and transient PL measurements, the samples were excited using a Laser-export Co. Ltd., frequency tripled, pulsed Nd:YAG laser at 3.49 eV (355 nm) and at 2.33 eV (532 nm) with 1 kHz repetition rate and detected respectively using an Hamamatsu Mini-Spectrometer and an Oriel Instrument Cornerstone 1/4 m monochromator coupled with a Hamamatsu UV–VIS photomultiplier tube. Cryogenic Measurements were performed in a liquid nitrogen-cooled Oxford Instruments Limited cryostat.

### DFT: comparison of monoclinic and cubic Pb_3_S_2_Cl_2_ structures

To assess the impact of the cubic symmetry breaking on the stability of Pb_3_S_2_Cl_2_ phase, we compared by DFT calculations the stability of the monoclinic Pb_3_S_2_Cl_2_ structure that we solved with that of a hypothetic cubic Pb_3_S_2_Cl_2_ structure. We performed this investigation on the corresponding 2 × 2 × 2 supercells at the gamma point. In particular, we relaxed both atomic positions and cell parameters at the DFT level using the PBE exchange−correlation functional^57^, and a double-ζ basis set plus polarization functions (DZVP) on all atoms as implemented in the CP2K 8.1 package^58^. Scalar relativistic effects were incorporated as effective core potentials in the basis set. With this approach, we determined that the monoclinic Pb_3_S_2_Cl_2_ 2 × 2 × 2 supercell is more stable than the prototypal cubic one by 164 kcal/mol.

### DFT: calculations on NCs models

Both the structural relaxation and the electronic structure calculations of atomistic NCs models were carried out at the DFT/PBE/DZVP level of theory with CP2K. In order to identify any surface localized states that could trap charge carriers, we also computed the Inverse Participation Ratio (IPR) for chalcohalide and perovskite, both in the form of free-standing NCs and in the heterostructure. The IPR, as also demonstrated for other NCs^26,59^, quantifies the orbital localization of a given molecular orbital and it is defined as:

$${{{{{{\rm{IPR}}}}}}}_{i}=\frac{{\sum }_{\alpha }{|{P}_{\alpha ,i}|}^{4}}{({\sum }_{\alpha }{{|{P}_{\alpha ,i}|}^{2}})^{2}}$$

Here, *P*_*α,i*_ represents the weight of molecular orbital *i* on a given atom *α* expanded in an atomic orbital basis. For finite systems, the IPR provides an estimate of the number of atoms that contribute to a given electronic state *i*. It can range from the inverse of the number of atoms in the system (when the wave function is distributed equally over all atoms in the system) to 1 in the case of states localized on single atoms. In other terms, IPR values very close to 0 identify delocalized states.

## Supplementary information

Supplementary Information

Supplementary Data 1

## Data Availability

The data obtained from 3D-ED, X-ray diffraction, HRSTEM column intensity analysis, optical spectroscopy, particle size distribution analysis, the results of the DFT calculations, and the structure models generated in this study have been deposited in the Zenodo database under accession code 10.5281/zenodo.6430001. The data obtained from X-ray diffraction, HRSTEM column intensity analysis, optical spectroscopy, particle size distribution analysis, and the results of the DFT calculations generated in this study are provided in the Source Data file. Crystallographic data for the structures reported in this Article have been deposited at the Cambridge Crystallographic Data Centre, under deposition numbers CSD 2181723 (Pb_3_S_2_Cl_2_), 2181721 (Pb_4_S_3_Br_2_) and 2181722 (Pb_4_S_3_I_2_). Copies of the data can be obtained free of charge via https://www.ccdc.cam.ac.uk/structures/. An atomistic model of the Pb_4_S_3_Cl_2_/CsPbCl_3_ epitaxial interface is provided as a Supplementary Data file (Supplementary Data 1.xyz). Source data are provided with this paper.

## Source data

Source Data
