# Supplementary material for: Halide perovskites as disposable epitaxial templates for the phase-selective synthesis of lead sulfochloride nanocrystals
Source: Nat Commun. 2022 Jul 8;13:3976. doi: 10.1038/s41467-022-31699-1 (PMC9270429; doi:10.1038/s41467-022-31699-1)
Supplement: Supplementary file 1 — Supplementary Information [file 41467_2022_31699_MOESM1_ESM.pdf]

Supplementary Information for

Halide Perovskites as Disposable Epitaxial Templates for the Phase-Selective Synthesis of Lead Sulfochloride Nanocrystals

Stefano Toso[a,b‡], Muhammad Imran[a‡*], Enrico Mugnaioli[c], Anna Moliterni[d*], Rocco Caliandro[d], Nadine J. Schrenker[e], Andrea Pianetti[f], Juliette Zito[a,g], Francesco Zaccaria[a], Ye Wu[a], Mauro Gemmi[c*], Cinzia Giannini[d], Sergio Brovelli[f*], Ivan Infante[a*], Sara Bals[e*], Liberato Manna[a*]

[a] Department of Nanochemistry, Istituto Italiano di Tecnologia, Via Morego 30, 16163 Genova, Italy

[b] International Doctoral Program in Science, Università Cattolica del Sacro Cuore, 25121 Brescia, Italy

[c] Electron Crystallography, Center for Materials Interfaces, Istituto Italiano di Tecnologia, Viale Rinaldo Piaggio 34, 56025 Pontedera, Italy

[d] Istituto di Cristallografia – Consiglio Nazionale delle Ricerche (IC–CNR), Via Giovanni Amendola 122/O, 70126 Bari, Italy

[e] Electron Microscopy for Materials Science (EMAT) and NANOlab Center of Excellence, University of Antwerp, Groenenborgerlaan 171, 2020 Antwerp, Belgium

[f] Dipartimento di Scienza dei Materiali, Università degli Studi di Milano-Bicocca, Via Roberto Cozzi 55, 20125 Milano, Italy

[g] Dipartimento di Chimica e Chimica Industriale, Università degli Studi di Genova, Via Dodecaneso 31, 16146 Genova, Italy

‡ These authors contributed equally

***Corresponding authors:** muhd.imran.phd@gmail.com ; annagrazia.moliterni@ic.cnr.it ; mauro.gemmi@iit.it ; sergio.brovelli@unimib.it ; ivan.infante@iit.it ; sara.bals@uantwerpen.be ; liberato.manna@iit.it

Contents

Supplementary Discussion 2

1. Synthesis of sulfohalide nanocrystals ($Pb_3S_2Cl_2$, $Pb_4S_3Br_2$, $Pb_4S_3I_2$) 2
2. Structural characterization of $Pb_3S_2Cl_2$ nanocrystals 5
3. Computational DFT modelling 13
4. Structure refinement of $Pb_4S_3X_2$ nanocrystals and comparison with $Pb_3S_2Cl_2$ 15
5. Models of $Pb_4S_3Cl_2/CsPbCl_3$ heterostructures 19
6. Synthesis and characterization of $Pb_4S_3Cl_2/CsPbCl_3$ heterostructures 21
7. Etching of heterostructures and characterization of $Pb_4S_3Cl_2$ nanocrystals 24
8. Spectroscopic characterization 27

Supplementary References 29

Supplementary Discussion

1. Synthesis of sulfohalide nanocrystals ($Pb_3S_2Cl_2$, $Pb_4S_3Br_2$, $Pb_4S_3I_2$)

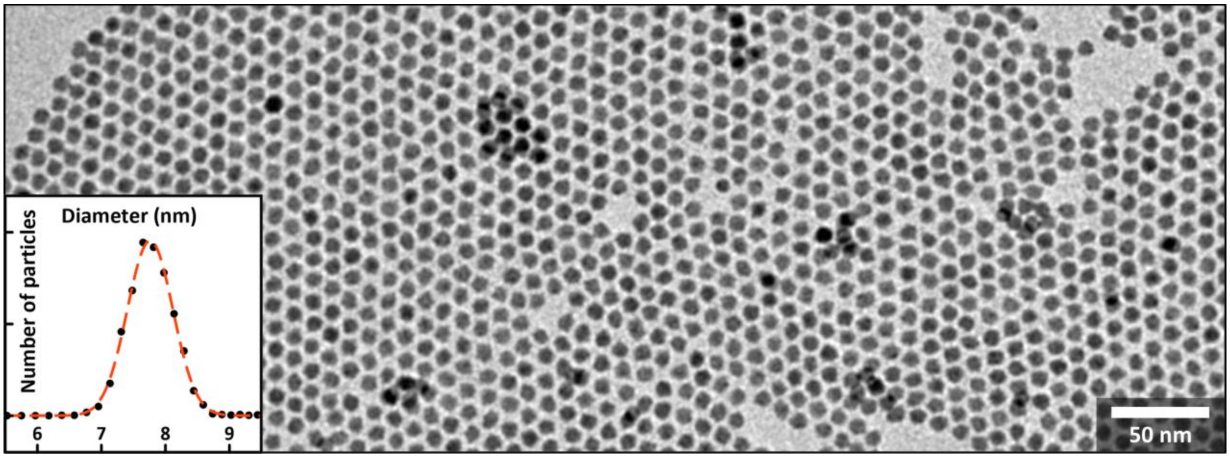

Supplementary Figure 1: TEM image of as-synthesised $Pb_3S_2Cl_2$ nanocrystals (NCs). The NCs size distribution (inset) was measured using the software FIJI-ImageJ[1] by applying the method described in the Supplementary Information of Ref. 2 on a total of 5252 NCs. Source data are provided as a Source Data file.

Accretion of larger $Pb_3S_2Cl_2$ NCs. Larger $Pb_3S_2Cl_2$ NCs were obtained through a seeded growth approach, consisting in reacting crude $Pb_3S_2Cl_2$ NCs obtained by direct synthesis with a stock solution of $PbCl_2$ and $Pb(SCN)_2$ at 170°C. During the dropwise addition of the stock solution, the heat decomposed $Pb(SCN)_2$. This acted as a source of free sulfur that, reacting with the $PbCl_2$ dissolved in solution, contributed to the accretion of $Pb_3S_2Cl_2$ NCs. The progress of the reaction was tracked by taking aliquots of the reaction mixture at T = 0h, 1h, 2h, 4h, and 5h:15min (reaction halted), which were analyzed by TEM (Supplementary Figure 2). Preliminary tests performed by using equimolar amounts of $PbCl_2$ and $Pb(SCN)_2$ resulted in the formation of large amounts of an impurity tentatively identified as platelets of $(OLA)_2PbCl_4$ (Supplementary Figure 3a, orange plot), which could not be removed and made the reaction product unsuitable for further studies. Increasing the relative amount of $Pb(SCN)_2$, that corresponds to the reaction conditions described in the Methods section of the Main Text, prevented the formation of such impurity and resulted instead in the nucleation of PbS impurities starting from ~4h into the process. Those however grew significantly larger than the $Pb_3S_2Cl_2$ NCs (Supplementary Figures 2f and 3b, the Selected Area Electron Diffraction (SAED) integrated pattern is shown in the Supplementary Figure 3a, blue plot), and could be quantitatively removed by filtering the NCs suspended in hexane with a 0.2 µm PTFE syringe filter and discarding any residual sediment. Indeed, the XRD analysis of the filtered product shows no trace of signals compatible with PbS (Supplementary Figure 3a, black plot).

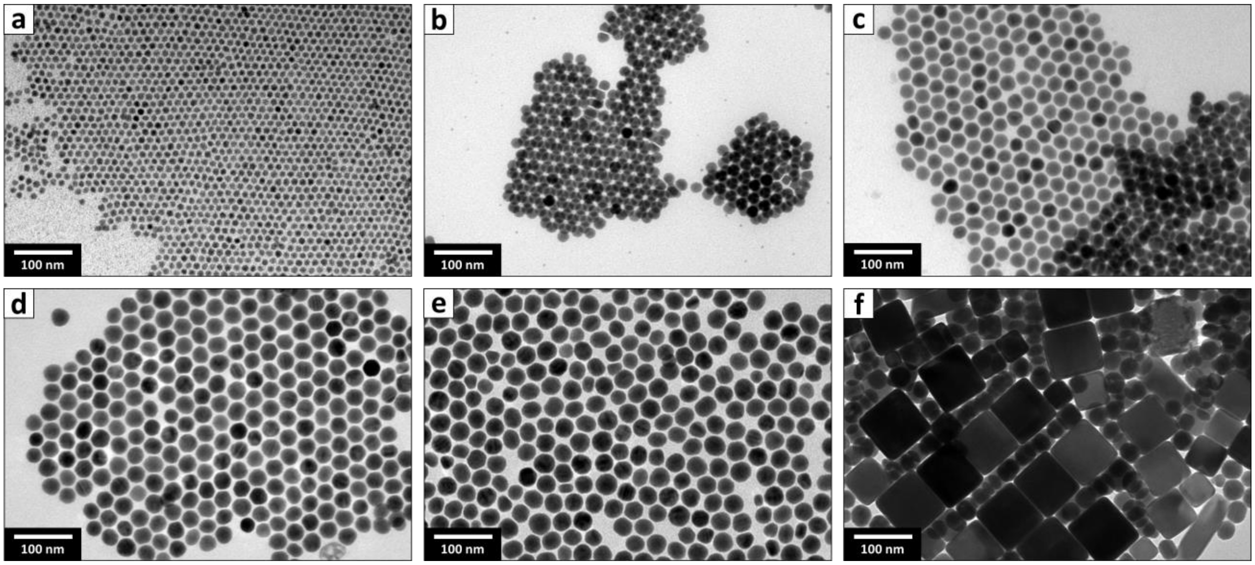

Supplementary Figure 2: Seeded growth of $Pb_3S_2Cl_2$ NCs. Aliquots of the $Pb_3S_2Cl_2$ NCs accretion reaction mixture taken a) at the start of the process, and after b) 1h, c) 2h, d) 4h, e) 5h:15min (reaction stopped). f) Large PbS impurities formed during the process, as found in the 5h:15min sample (e).

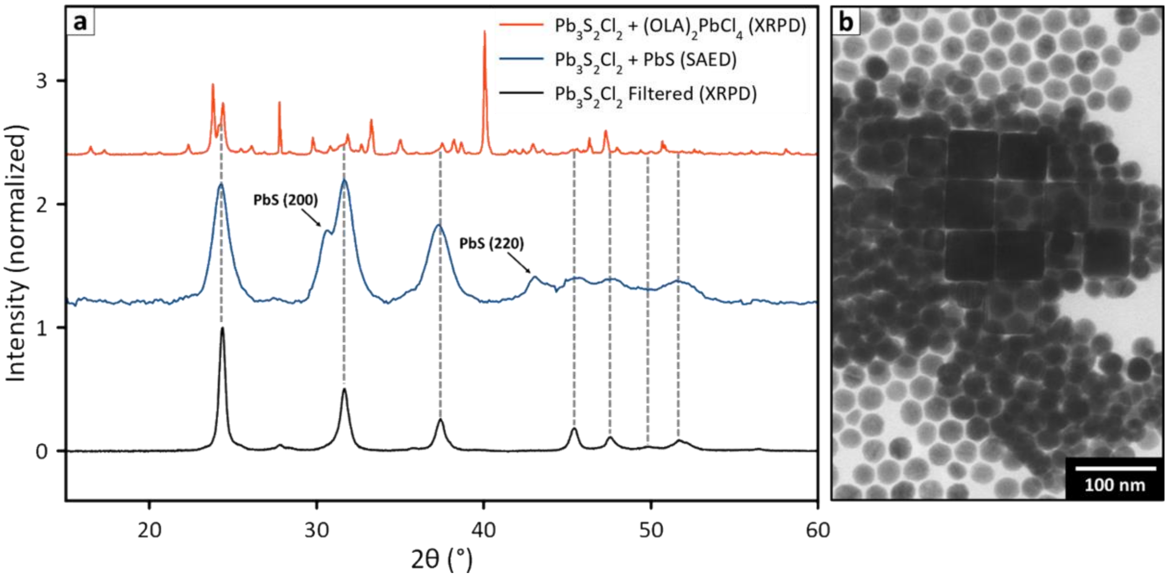

Supplementary Figure 3: Diffraction patterns of large $Pb_3S_2Cl_2$ NCs obtained by seeded growth. a) Diffraction patterns of samples obtained by accreting $Pb_3S_2Cl_2$ NCs seeds with equimolar amounts of $Pb(SCN)_2$ and $PbCl_2$ (XRPD data, orange plot) and with an excess of $Pb(SCN)_2$ over $PbCl_2$ (SAED data, blue plot). The XRPD pattern of the filtered sample (black plot) shows no sign of PbS impurities. b) Area of the sample on which the SAED data plotted in panel (a) were acquired, showing large cuboidal PbS crystals oriented along the <100> zone axis. Source data are provided as a Source Data file.

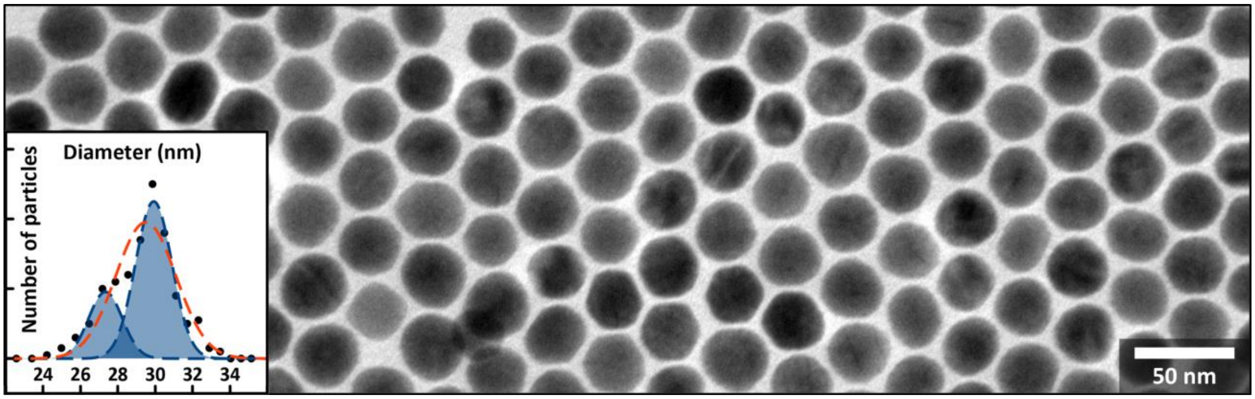

Supplementary Figure 4: TEM image of $Pb_3S_2Cl_2$ NCs after seeded growth and purification. The NCs size distribution (inset) was estimated using the software FIJI-ImageJ[1] by adapting the method described in Ref. 2 on a total of 250 NCs. After the accretion, the size distribution in the sample appears to be bimodal (two blue gaussians in the inset), but it is still roughly captured by a single gaussian distribution (red in the inset). Source data are provided as a Source Data file.

Supplementary Table 1: SEM-EDX compositional analysis on $Pb_3S_2Cl_2$ NCs. The analysis was performed on a sample of dried and pressed NCs powders.

	**Pb (at. %)**	**S (at. %)**	**Cl (at. %)**
**Region 1**	43.55	26.93	29.52
**Region 2**	45.32	26.65	28.03
**Region 3**	46.68	25.42	27.90
**Average ± st. dev.**	46.0 ± 1.0	26.3 ± 0.8	28.5 ± 0.9

2. Structural characterization of $Pb_3S_2Cl_2$ nanocrystals

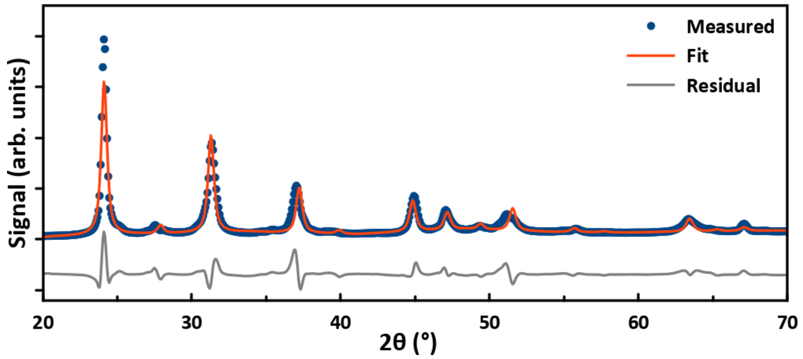

Supplementary Figure 5: Attempt to fit the $Pb_3S_2Cl_2$ NCs pattern based on the $Pb_3Se_2Br_2$ cubic prototype. The XRPD experimental data (blue) and fit profile (orange) are plotted in the Cu-K_α 2θ scale to ease the comparison with patterns collected with lab-grade instruments. In correspondance of the peaks (e.g. 2θ = 31.3° and 37.0°), the residual curve (grey) clearly indicates that the simulated peak position is off with respect to the experimental one. However, the simulated peak at 31.3° falls at lower angles if compared with the measurement (i.e., longer periodicities), while the peak at 37.0° falls at higher angles (i.e., shorter periodicities). This kind of mismatch cannot be complensated by simply resizing a cubic unit cell, and requires to take into consideration a lower symmetry structure. Source data are provided as a Source Data file.

Single-Nanocrystal 3D-ED experiments and ab-initio structure solution of $Pb_3S_2Cl_2$. 3D Electron Diffraction (3D-ED)[3,4] was exploited to solve the structure of $Pb_3S_2Cl_2$ NCs. 3D-ED data were collected from roughly spherical NCs with a diameter of ~25 nm, prepared as described in the Supplementary Discussion, section 1. During the data acquisition the electron beam was stationary, while the sample was tilted in fixed steps of 1° for a maximum total range up to 110°. In fact, diffraction data acquired with a precessing beam[5,6] were blurred due to the small size of the particles, and thus were not suitable for the 3D reconstruction. Therefore, only the data acquired with a stationary beam were used, with a consequent deterioration of the accuracy on the integrated intensities of reflections. The data were first analyzed using ADT3D[7] for the cell and space group determination, which resulted in a primitive triclinic unit cell with parameters $a \approx b$ = 7.8(2) Å; c = 7.7(2) = Å and α = 109.7(5)°, β = 109.1(5)°, γ = 109.4(5)°. This unit cell is compatible, within the rather large experimental error, with several higher-symmetry unit cell choices: I-centered cubic (a = 9.0 Å, Supplementary Figure 6a), primitive hexagonal (a = 12.8 Å; c = 7.7 Å), trigonal (a = 7.8 Å; α = 109.4°), or C-centered monoclinic ($a \approx b$ = 12.7 Å, c = 7.7 Å, β = 144.3°, Supplementary Figures 6b-d).

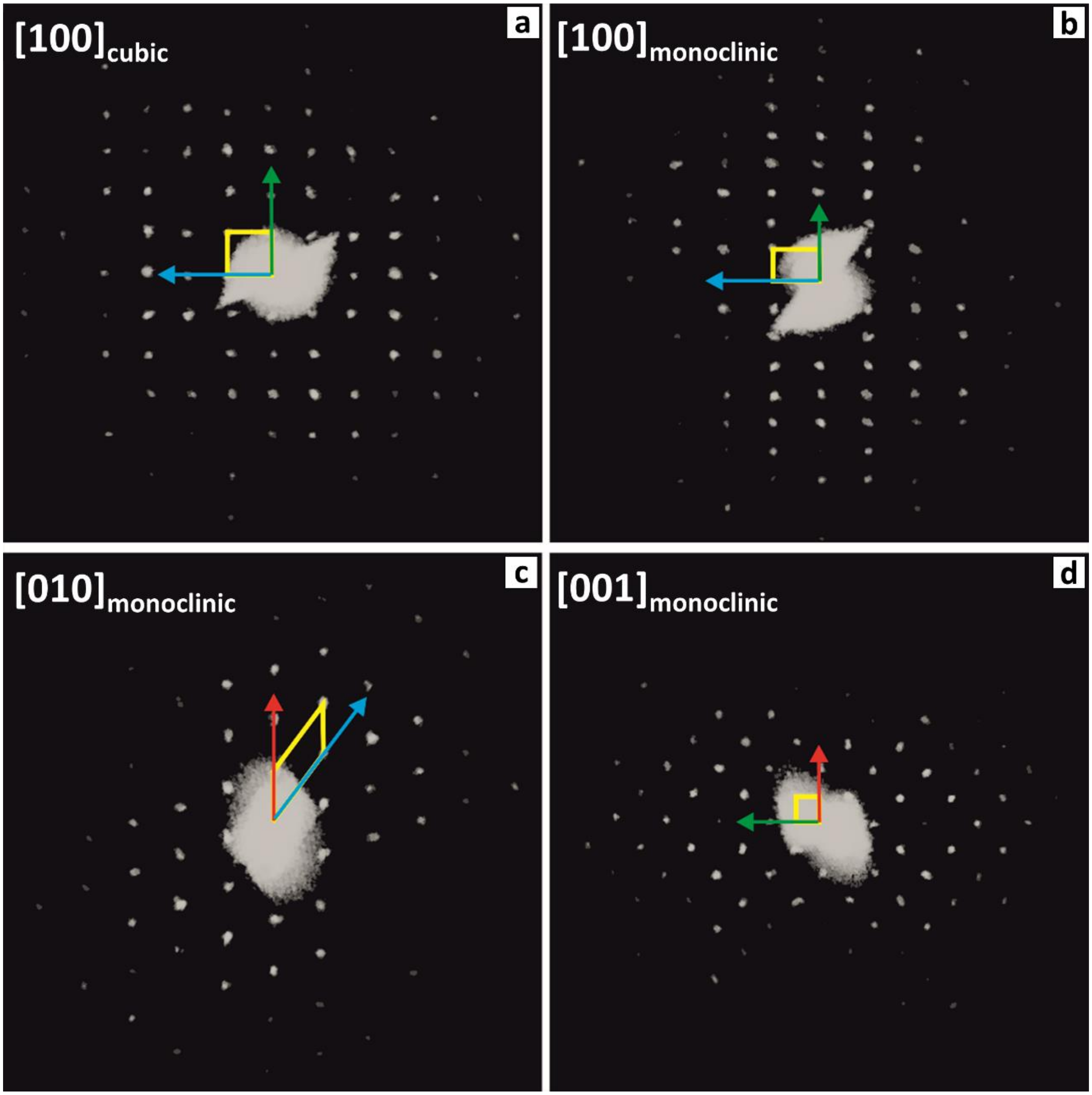

Supplementary Figure 6: 3D-ED diffraction data from a single $Pb_3S_2Cl_2$ NC. The data are shown a) along the [100] projection of the pseudocubic cubic lattice and b-d) along the [100], the [010] and the [001] projections of the possible monoclinic lattice. In all the panels, the reciprocal cell edges are shown in yellow, the projections of the *a** vector are sketched in red, the projections of the *b** vector are sketched in green and the projections of the *c** vector are sketched in blue. The panels relative to the monoclinic lattice (b-d) are indexed accordingly to the unit cell choice proposed by the indexation software, prior to the standardization of the unit cell that we later performed on the refined $Pb_3S_2Cl_2$ structure model.

Due to the small size of the NCs, the resolution of the collected datasets was limited to about 1.0-1.5 Å. Nevertheless, we attempted an *ab-initio* structure solution. The intensity integration for the structure determination was performed with in-house MATLAB routines, while the solution was performed using direct methods implemented in the software SIR2014.[8] The data were treated within the kinematical approximation: $I_{hkl} \propto F^2_{hkl}$. The first solution attempt was performed in space group *I*-43d, the same reported for $Pb_3Se_2Br_2$,[9] and produced a model with comparable atom distribution and connectivity. This evidence strengthened the hypothesis that $Pb_3S_2Cl_2$ was indeed derived from such prototypal structure. An analogue solution was obtained in the space group $I2_13$ (Supplementary Figure 7a), a subgroup of *I*-43d that allows to assign different crystallographic positions to sulfur and chlorine (while in the *I*-43d they randomly occupy the same position). A second attempt, performed in the *C*c space group, produced a similar, yet distorted structure (Supplementary Figure 7b). In all the cases, the Pb atoms could be unambiguously identified, while Cl and S could not be distinguished because of the very similar scattering factors.

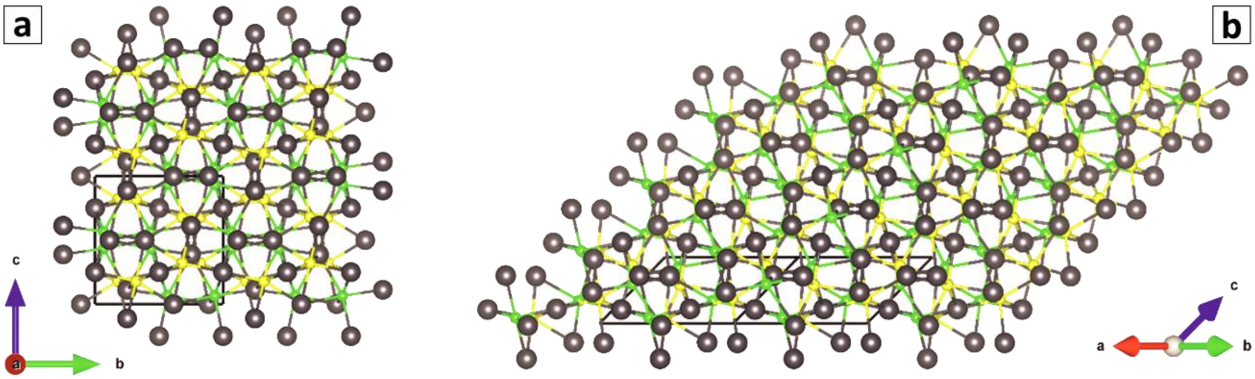

Supplementary Figure 7: $Pb_3S_2Cl_2$ structure solutions obtained from 3D-ED data. The two solutions were obtained a) in the space group $I2_13$ and b) in the space group *C*c. The sulfur and chlorine species are tentatively assigned, due to the insufficient difference in the scattering factors. The unit cell choice is shown prior to the standardization that we performed later on the refined $Pb_3S_2Cl_2$ model (black lines). Atoms color code: Pb = grey; S = yellow; Cl = green.

Ab initio structure solution of $Pb_3S_2Cl_2$ from X-Ray Powder Diffraction (XRPD). The main sources of uncertainty in the 3D-ED solution were the low resolution of the dataset and the potential inaccuracies of the integrated intensities of reflections, due both to the secondary scattering phenomena, which were neglected by treating data within the kinematical approximation, and mostly to the inability to acquire data in precession mode. For this reason, we relied on XRPD data collected at the 28ID-2 beamline of the National Synchrotron Light Source (NSLS-II) of the Brookhaven National Laboratory to reattempt an ab-initio solution and then refine the structure of $Pb_3S_2Cl_2$ NCs.

The ab initio structure solution by XRPD data was carried out using EXPO2014,[10] a software package that implements all the steps of the process: indexing, space group determination, full pattern decomposition, structure solution, structure optimization, and Rietveld refinement. The first two steps (i.e., indexing and space group determination) were highly challenging for our specific sample, as the expectedly minor distortions from the prototypal $Pb_3Se_2Br_2$ cubic structure[9] and the severe size-related broadening of the diffraction features produced an extreme overlap of the reflections. This in turn caused the failure of the indexing routine, and introduced unavoidable errors in the intensity integration, that led the automatic space group determination process to produce incorrect results. Hence, the indexation and space group determination steps were performed by drawing from the information provided by 3D-ED. The following monoclinic cell parameters, initially determined by 3D-ED data and further optimized by Pair Distribution Function (PDF) analysis, were supplied as input values to EXPO2014:

$$a = 12.54099,\ b = 12.77492,\ c = 7.59766\ \text{Å},\ \beta = 144.629°$$

The space group *C*c, suggested by 3D-ED, was used to estimate the integrated intensity of reflections and consequently also the structure factor moduli required for solving the crystal structure by Direct Methods. Among the possible cells identified by 3D-ED, the *C*c monoclinic choice was preferred based on the best agreement between the calculated and the experimental positions of reflections. The application of Direct Methods produced twenty sets of phases, ranked according to a combined figure of merit. An automatic procedure was applied to select the best candidate from each set based on the agreement factor between the observed and calculated structure factor moduli. Remarkably, the first ten models by rank were in great overlap with each other and with the structure determined by 3D-ED, the major differences being due to the ambiguous assignment of the sulfur and chlorine positions. Out of these 10 solutions, one was selected *via* the graphic user interface of EXPO2014 according to structural chemistry considerations (e.g., adjacency of cations and anions, homogeneity of the coordination environment for atoms of the same element, etc.). This selected model then underwent a first optimization through the Rietveld refinement routine implemented in the EXPO2014 package.

Combined XRPD + PDF refinement of the $Pb_3S_2Cl_2$ NCs structure. The structure model obtained from the ab-initio XRPD solution was refined by combining the analysis of XRPD and PDF data, both collected at the 28ID-2 beamline at NSLS-II. It is important to note that the two datasets, XRPD and PDF, are independent from each other as the position of the detector was changed for the two experiments.

The PDF profile was refined by using the programs PDFGUI[11–13] and DiffPy-CMI[16]. The fit was performed for interatomic distances (*r*) above 1.5 Å, to avoid finite-size artifacts in the low *r* range, and up to 50 Å, with a step of 0.05 Å. At first, Python scripts were used to perform an automatic refinement where all the parameters were optimized one at a time, while keeping constant all the others. The parameters were refined in the following order: scale factor, lattice parameters, Q_{broad}, (peak broadening from increased noise intensity at high Q), δ_1 (coefficient for the $1/r$ contribution to the peak sharpening), atomic displacement parameters, and atomic coordinates. During the refinement, the crystal symmetry was exploited to constrain lattice, displacement parameters, and atomic coordinates. The refinement ran for a total of 20 cycles: during the first 10, the atomic displacement parameters were kept isotropic, while they were set to anisotropic in the last 10 cycles.

As a second step, the structure was refined interactively starting from the model resulting from the first step. At this stage, the fitting parameters were refined to treat structural distortions and outlier parameters. The structure optimization was carried out by alternating refinement procedures in the direct space (PDF) and in the reciprocal space (XRPD, through the EXPO2014 refinement routine). We choose to alternate PDF and XRPD refinements to avoid overfitting and local minima, exploiting the fact that the PDF and XRPD profiles we collected were not related by a simple Fourier transform, since the position of the detector was different for the two experiments. This allowed optimizing the experimental setup for both measurements, hence allowing us to make the best use of complementary information in both the direct and reciprocal spaces to drive the structure refinement.

Supplementary Figure 8 shows the asymmetric unit of the refined structure and its local environment. The observed, calculated and difference profiles resulting from the last cycle of the Rietveld and PDF refinements are shown in Figure 2d of the Main Text. The outcome of the refinement is summarized in Supplementary Table 2 (XRPD) and Supplementary Table 3 (PDF).

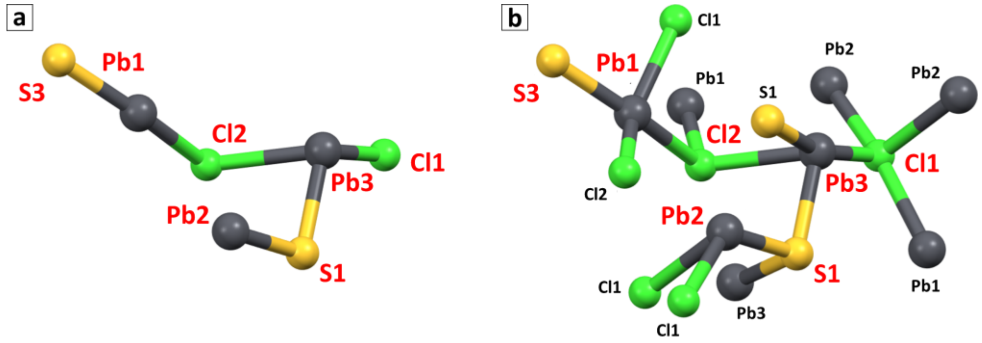

Supplementary Figure 8: Asymmetric unit of $Pb_3S_2Cl_2$. A view of a) the asymmetric unit of $Pb_3S_2Cl_2$ (red label atoms) and b) its local environment (black label atoms) as obtained from the last cycle of Rietveld refinement. Atoms color code: Pb = grey; S = yellow; Cl = green.

Supplementary Table 2. Results of the XRPD refinement of $Pb_3S_2Cl_2$ NCs. Goodness of fit and unit cell parameters obtained during the last cycle of the XRPD fit for $Pb_3S_2Cl_2$ NCs. R_p is the agreement factor between observed and calculated profile, R_{wp} is the weighted-profile reliability parameter, χ^2 is the chi-squared value, GoF is the Goodness of Fit.

	**XRPD-Rietveld refinement of $Pb_3S_2Cl_2$**
R_p	0.082
R_{wp}	0.111
χ^2	1.236
**GoF**	1.112
***a, b, c* (Å)**	12.595(3), 12.771(3), 7.6624(14)
β **(°)**	144.547(8)

Supplementary Table 3: Results of the PDF refinement of $Pb_3S_2Cl_2$ NCs. Goodness of fit and unit cell parameters obtained during the last cycle of the PDF fit for $Pb_3S_2Cl_2$ NCs. R_w is the weighted agreement factor between observed and calculated PDF, δ_1 is the coefficient for 1/*r* contribution to the peak sharpening, Q_{broad} describes the peak broadening from increased intensity noise at high *Q*, $SP_{diameter}$ is the particle diameter for PDF shape damping function.

	**PDF refinement of $Pb_3S_2Cl_2$**
R_w	0.155
δ_1	2.27
Q_{broad}	0.004
$SP_{diameter}$ **(Å)**	80.3
***a, b, c* (Å)**	12.549, 12.783, 7.603
β **(°)**	144.726

Supplementary Figure 9 compares the model obtained at the end of the combined XRPD+PDF refinement procedure with that originally provided by 3D-ED. The two structures are in remarkable overlap, being characterized by a Root Mean Square Deviation of only 0.369 Å ($\mathrm{RMSD} = \sqrt{\sum_i d_i^2 / N_{au}}$, where d_i are the distances between couples of corresponding i^{th} atoms in the two compared models, and N_{au} is the total number of atoms in the asymmetric unit).

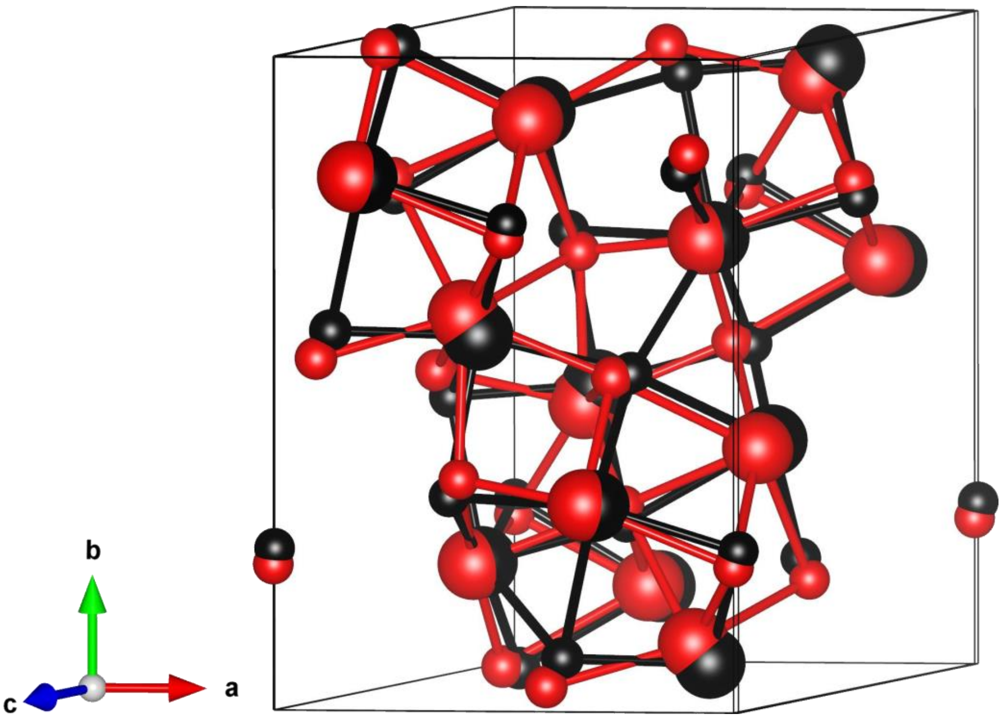

Supplementary Figure 9: $Pb_3S_2Cl_2$ structure models from 3D-ED and XRPD+PDF compared. The $Pb_3S_2Cl_2$ structure models obtained by 3D-ED (black) and by XRPD+PDF (red) demonstrate a strong overlap. In this image, the elements are differentiated by size (Pb = large atoms; S/Cl = small atoms). Both structures are shown after applying the crystal data standardization routine implemented in VESTA.[14]

Crystal data standardization. As a final step after the refinement, we applied the crystal data standardization routine implemented in the software VESTA[14] to produce the $Pb_3S_2Cl_2$ structure model that is shown in Figure 2e of the Main Text and in the Supplementary Figure 9. The procedure simply results in a different but equivalent unit cell choice, and does not alter the resulting structure at all. The standardized structure model has been deposited at the Cambridge Crystallographic Data Centre, under deposition numbers CCDC 2181723, while the model prior to the standardization is available in the Zenodo database under the accession code https://doi.org/10.5281/zenodo.6430001.

HAADF-STEM imaging of $Pb_3S_2Cl_2$ NCs. The structure proposed for $Pb_3S_2Cl_2$ was checked against the High-Angle Annular Dark-Field Scanning Transmission Electron Microscopy (HAADF-STEM) images of $Pb_3S_2Cl_2$ NCs, as summarized in the Supplementary Figure 10. As expected, the structure model we obtained could be overlaid with the atomic-resolution images, and the Fast Fourier Transforms (FFTs) of the HAADF-STEM images could be indexed according to our proposed structure.

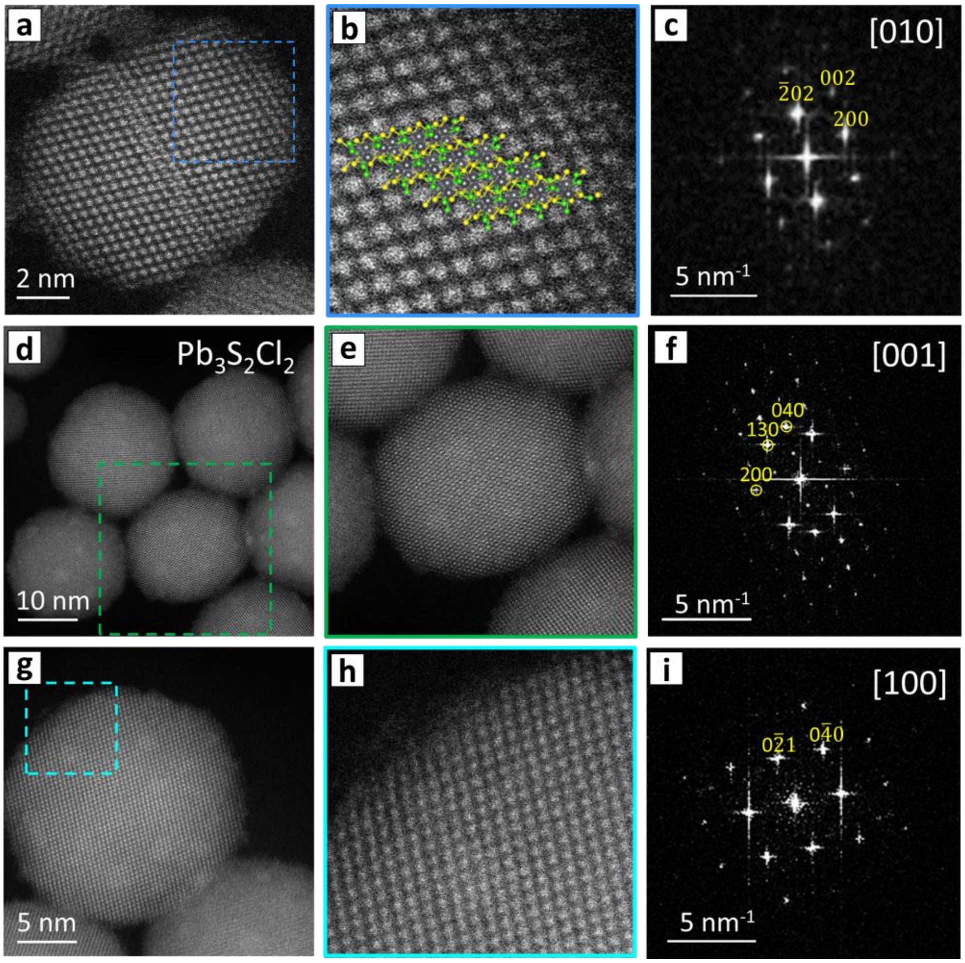

Supplementary Figure 10: HAADF-STEM characterization of $Pb_3S_2Cl_2$ NCs. Colloidal NCs of $Pb_3S_2Cl_2$ a-c) as-synthesized and d-i) after the accretion process. a) Image of a $Pb_3S_2Cl_2$ NC. b) Close-up of the area highlighted in the blue square in panel (a). A projection of the crystal structure along the [010] zone axis is overlayed. c) Fast Fourier transform (FFT) of the particle in panel (a), revealing that the $Pb_3S_2Cl_2$ NC is oriented along the [010] zone axis. d) Overview image of NCs after the accretion process. e) Close-up of the area highlighted in green in panel (d). f) FFT of the NC in panel (e) revealing an orientation along the [001] zone axis. (g) NCs oriented along the [100] zone axis, as revealed by the image in panel (h) and the FFT in panel (i). All the FFT signals are indexed according to the model prior to the crystal data standardization procedure. Atoms color code: Pb = grey; S = yellow; Cl = green.

3. Computational DFT modelling

DFT analysis of $Pb_3S_2Cl_2$ and $Pb_4S_3Cl_2$ NCs. To provide an atomistic insight into free-standing $Pb_3S_2Cl_2$ NCs, we first prepared the $Pb_3S_2Cl_2$ NC model illustrated in the Supplementary Figure 11a by cutting the monoclinic bulk structure (Figure 2e of the Main Text) into a charge-balanced sphere of about 4.2 nm in diameter with a nearly stoichiometric Pb:S:Cl=3:2:2 ratio. The relaxation of the model substantially preserved the monoclinic phase. We then computed the fractional Density of States (DOS) and the Inverse Participation Ratio (IPR), shown in the Supplementary Figure 11b. This revealed the dominant contribution of Pb (in black) and S (in yellow) to the conduction band (CB) and valence band (VB) states, and the emergence of a large number of localized trap states, as indicated by IPR values much larger than 0 at both the band edges. We also extrapolated the actual intake of the CB and VB edges at about 1.8 eV by filtering out the trap states with IPR above 0.03. An equivalent approach was applied to the free-standing $Pb_4S_3Cl_2$ NCs. Here, we prepared the computational model by cutting in half a charge-balanced $Pb_4S_3Cl_2$ sphere of about 4.2 nm in diameter, as illustrated in the Supplementary Figure 11c. Notably, the electronic features of this system, reported in the Supplementary Figure 11d, closely resemble those of the free-standing $Pb_3S_2Cl_2$ NC, with the band edges being dominated by the Pb and S contributions and fully defined by trap states. In this case, the actual value of the band gap was extrapolated at about 1.6 eV.

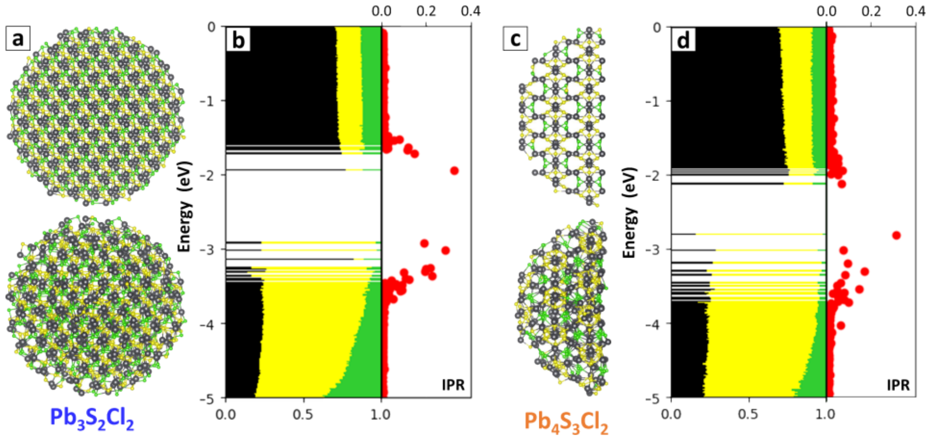

Supplementary Figure 11: DFT calculations on $Pb_3S_2Cl_2$ and $Pb_4S_3Cl_2$ NCs. a) Atomistic model of a $Pb_3S_2Cl_2$ NC that was built to perform DFT calculations, before (top) and after (bottom) the structure optimization. b) Fractional DOS (color-coded by element) and IPR (in red) of the $Pb_3S_2Cl_2$ NC model. c) Atomistic model of a $Pb_4S_3Cl_2$ NC that was built to perform DFT calculations, before (top) and after (bottom) the structure optimization. The shape is hemispherical because the same domain was used as a part of the $Pb_4S_3Cl_2/CsPbCl_3$ heterostructure model (Supplementary Figure 12). d) Fractional DOS and IPR of the $Pb_4S_3Cl_2$ NC model. The two DOS panels are also shown in Figure 5d of the Main Text. Atoms color code: Pb = black; S = yellow; Cl = green. Source data are provided as a Source Data file.

DFT analysis of $Pb_4S_3Cl_2/CsPbCl_3$ heterostructures. We then computed the $Pb_4S_3Br_2/CsPbCl_3$ heterostructure model shown in the Supplementary Figure 12. Remarkably, the starting alignment of Pb^{2+} ions across the epitaxial interface was spontaneously preserved after the structure relaxation. As shown by the computed DOS and IPR reported in Figures 5e of the Main Text, both band edges of the resulting heterostructure model are fully composed by trap states originating only from the $Pb_4S_3Cl_2$ domain. A closer look at the corresponding molecular orbitals, represented in Figure 5f of the Main Text, reveals that these states concentrate at the chalcohalide surface, similar to what we observed for $Pb_4S_3Br_2/CsPbCl_3$ heterostructures in the past.[15] In this case however, the first delocalized states at both the CB and the VB edges also appear to be dominated by the $Pb_4S_3Cl_2$ system (Figure 5e of the Main Text), suggesting a type-I energy level alignment.

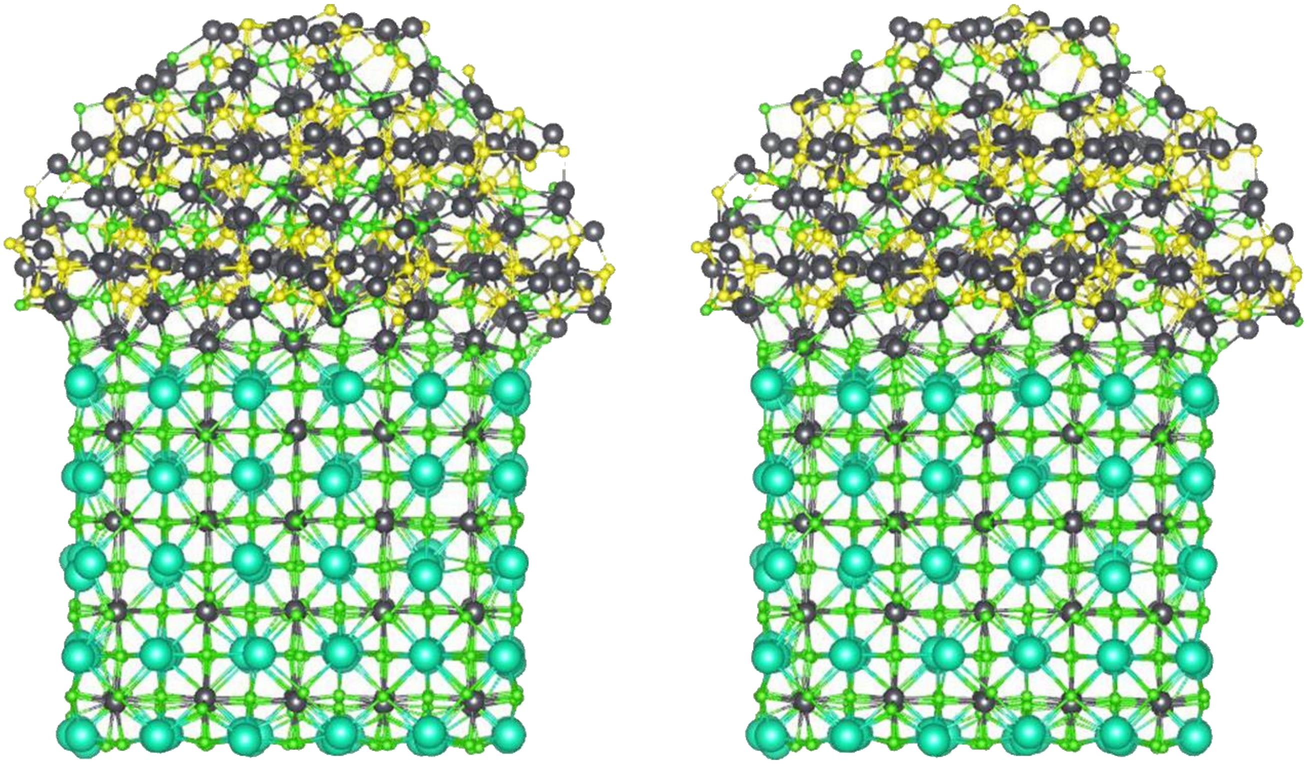

Supplementary Figure 12: $Pb_4S_3Br_2/CsPbCl_3$ heterostructure atomistic model. The atomistic $Pb_4S_3Br_2/CsPbCl_3$ heterostructure model built to perform DFT calculations is here shown before (left) and after (right) the structure optimization. The two models appear very similar because both the $Pb_4S_3Br_2$ and the $CsPbCl_3$ moieties were pre-optimized before assembling the heterostructure model. Atoms color code: Cs = cyan; Pb = grey; S = yellow; Cl = green.

4. Structure refinement of $Pb_4S_3X_2$ nanocrystals and comparison with $Pb_3S_2Cl_2$

Combined XRPD+PDF structure refinement of $Pb_4S_3Br_2$ and $Pb_4S_3I_2$ NCs. We refined the structure of $Pb_4S_3Br_2$ and $Pb_4S_3I_2$ NCs, that we already reported in our prior publication,[16] in order to better compare such phases with the $Pb_3S_2Cl_2$ structure that was solved in this work. In addition, we want to provide the community with more reliable structure models than those we reported by us in the past, as they were refined only on lab-grade XRPD data. Hence, at NSLS-II we collected XRPD and PDF data on $Pb_4S_3Br_2$ and $Pb_4S_3I_2$ NCs, and we refined both structures following a procedure analogue to that detailed in the Supplementary Discussion, section 2. The outcome of the analyses is summarized in the Supplementary Figures 13-14 and in Supplementary Table 4. The resulting structure models have been deposited at the Cambridge Crystallographic Data Centre, under deposition numbers 2181721 ($Pb_4S_3Br_2$) and 2181722 ($Pb_4S_3I_2$).

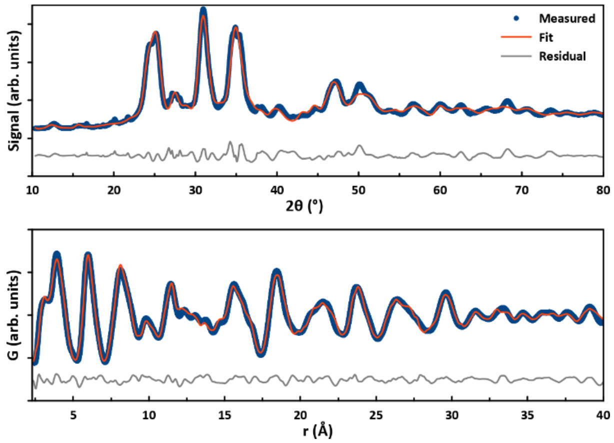

Supplementary Figure 13: Refined XRPD (top) and PDF (bottom) profiles of $Pb_4S_3Br_2$ NCs. The experimental data are plotted in blue, the fit profiles in orange and the residuals in grey. Source data are provided as a Source Data file.

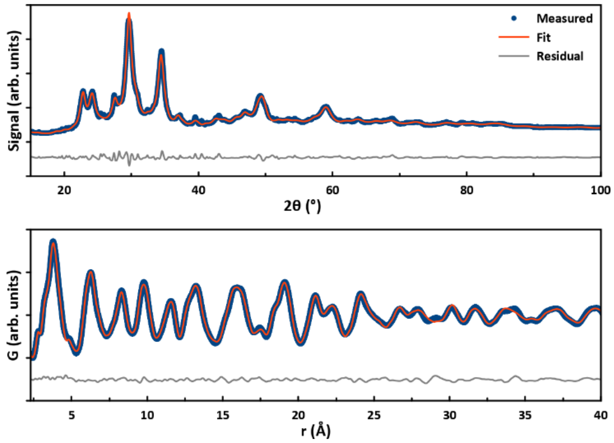

Supplementary Figure 14: Refined XRPD (top) and PDF (bottom) profiles of $Pb_4S_3I_2$ NCs. The experimental data are plotted in blue, the fit profiles in orange and the residuals in grey. Source data are provided as a Source Data file.

Supplementary Table 4: XRPD and PDF refinement parameters for $Pb_4S_3Br_2$ and $Pb_4S_3I_2$ NCs. For XRPD, R_p is the agreement factor between observed and calculated profile, R_{wp} is the weighted-profile reliability parameter, χ^2 is the chi-squared value, GoF is the Goodness of Fit. For PDF, R_w is the weighted agreement factor between observed and calculated PDF, δ_1 is the coefficient for 1/*r* contribution to the peak sharpening, Q_{broad} describes the peak broadening from increased intensity noise at high *Q,* $SP_{diameter}$ is the particle diameter for PDF shape damping function.

XRPD refinements			PDF refinement of $Pb_4S_3I_2$		
**Parameter**	**$Pb_4S_3Br_2$**	**$Pb_4S_3I_2$**	**Parameter**	**$Pb_4S_3Br_2$**	**$Pb_4S_3I_2$**
R_p	0.0583	0.0291	R_w	0.152	0.085
R_{wp}	0.0758	0.040	δ_1	-0.36	2.54
χ^2	4.8919	1.0958	Q_{broad}	0.042	0.031
**GoF**	2.2118	1.0468	**$SP_{diameter}$ (Å)**	73.6	62.0
a, b, c **(Å)**	8.178, 14.732, 8.095	8.227, 15.642, 8.248	***a, b, c*** **(Å)**	8.217, 14.624, 8.091	8.239, 15.619, 8.246

It is worth noting that the quality of the XRPD Rietveld fit for $Pb_4S_3Br_2$ is not optimal, and there are some discrepancies in the unit cell parameters as refined by XRPD and PDF. We attribute this to a combination of an intrinsically challenging fit for XRPD, where many relevant reflections overlap due to their small relative angular distance and to the intrinsic size-related broadening of diffraction features, plus some possible small deviations from the ideal orthorhombic symmetry in which the structure was refined. Those might reflect on the slight deviations between observed and calculated PDF profiles at large interatomic distances ($r > 40$ Å), and affect the overall quality of the XRPD fit. However, given the minor relevance of the $Pb_4S_3Br_2$ refinement in this work, we decided not to investigate further.

Comparison of $Pb_3S_2Cl_2$, $Pb_4S_3Br_2$, and $Pb_4S_3I_2$ structures. Supplementary Figure 15 compares the refined structures of the three compounds we examined, with a focus on the coordination polyhedra of each element. As discussed in the Main Text, the Pb and S atoms share similar coordination environments in all the three compounds. Conversely, Cl has a different coordination in $Pb_3S_2Cl_2$ (6 Pb atoms, distorted octahedral) than Br and I in $Pb_4S_3X_2$ (7 Pb atoms, pentagonal bipyramid), pointing to the influence of the halide ionic radius in determining which stoichiometry and structure is favored during the formation of free-standing lead sulfohalide NCs.

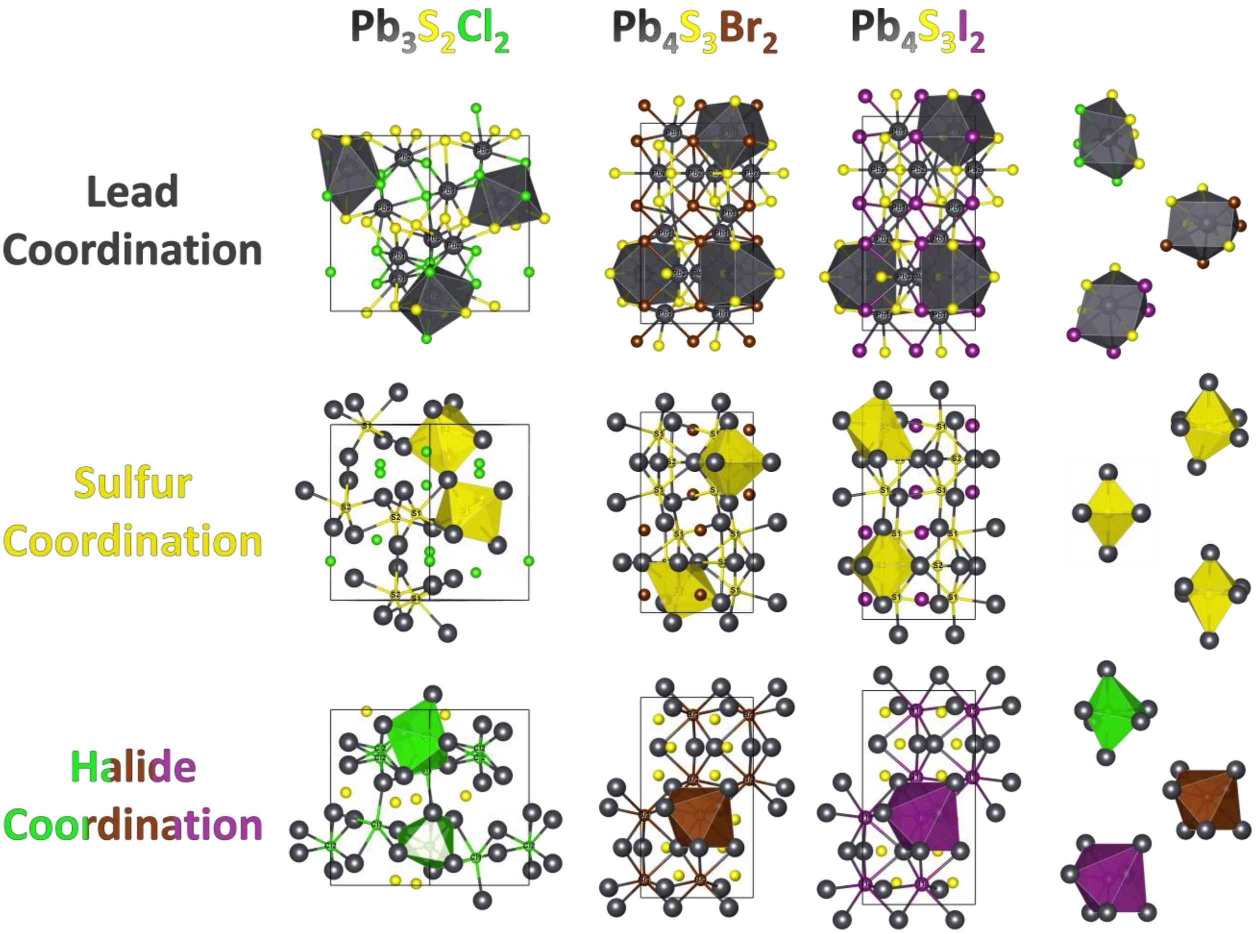

Supplementary Figure 15: Refined $Pb_3S_2Cl_2$, $Pb_4S_3Br_2$, and $Pb_4S_3I_2$ structures. The three structures are represented highlighting the coordination polyhedra of each non-equivalent crystallographic position. The column on the right compares the coordination polyhedra of lead (top), sulfur (middle) and halides (bottom) in the three compounds. The structures shown here for $Pb_4S_3Br_2$ and $Pb_4S_3I_2$ come from the last refinement cycle of the PDF analysis, that has the advantage of prioritizing the accuracy of the local coordination environment if compared to the Rietveld XRPD analysis. Atoms color code: Pb = grey; S = yellow; Cl = green; Br = brown; I = purple.

__*Comparison of $Pb_3S_2Cl_2$, $Pb_4S_3Br_2$, and $Pb_4S_3I_2$ PDF profiles.*__ Further insights on the role of the halide radii in $Pb_3S_2Cl_2$, $Pb_4S_3Br_2$ and $Pb_4S_3I_2$ come from interpreting their PDF profiles (Supplementary Figure 16) with the help of the structure models produced from the refinement. Indeed, the peaks corresponding to Pb-S distances correspond approximately to the same interatomic distance in all compounds (solid black arrow, Pb-S ~2.85 Å). Conversely, the Pb-X bonds (dashed arrow) expand from 2.9 Å in $Pb_3S_2Cl_2$ to 3.3 Å in $Pb_4S_3I_2$, consistently with the increasing ionic radii. Interestingly, the Pb-Pb distances (dotted arrow) follow an opposite trend, as they decrease from ~4.1 Å in $Pb_3S_2Cl_2$ to ~3.8 Å in $Pb_4S_3I_2$. Together, these two facts highlight how the expansion of the coordination environment around the halide atoms, captured by the elongation of the Pb-X bond lengths, forces the material to adopt a more compact structure for Br and I with respect to Cl. This is also captured by the calculated density, which raises from 6.95 g/cm^3 for $Pb_3S_2Cl_2$ to ~7.38 g/cm^3 for both $Pb_4S_3Br_2$ and $Pb_4S_3I_2$.

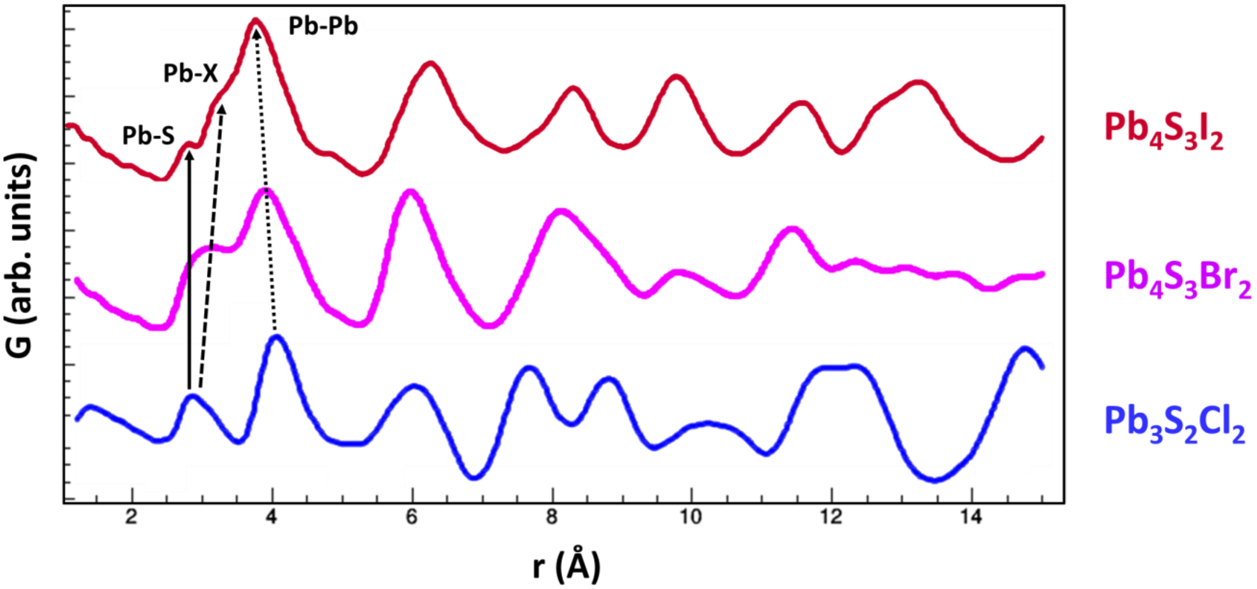

Supplementary Figure 16: PDF profiles of $Pb_3S_2Cl_2$, $Pb_4S_3Br_2$ and $Pb_4S_3I_2$ NCs compared. The evolution of the Pb-S, Pb-X and Pb-Pb distances is indicated by solid, dashed and dotted arrows, respectively. The profiles have been normalized, and were offset vertically by an arbitrary constant for representation purposes. The program RootProf[19] was used to elaborate and visualize the PDF profiles. Color code: $Pb_4S_3I_2$ = red, $Pb_4S_3Br_2$ = purple, $Pb_4S_3Cl_2$ = blue. Source data are provided as a Source Data file.

5. Models of $Pb_4S_3Cl_2$/$CsPbCl_3$ heterostructures

Cationic subnetwork in $Pb_4S_3Cl_2$/$CsPbCl_3$ heterostructures. The role of the cationic subnetwork in the formation of the $Pb_4S_3Cl_2$/$CsPbCl_3$ heterostructures, and its similarity with the Cs-Pb-X/$CsPbX_3$ epitaxial heterojunctions, can be better visualized by comparing its structure with those of $CsPb_2Cl_5$/$CsPbCl_3$ (left) and Cs_2PbCl_4/$CsPbCl_3$ heterostructures (Supplementary Figure 17). In all the three models the perovskite $CsPbCl_3$ moiety remains unchanged.

The first comparison highlights that both $Pb_4S_3Cl_2$ and $CsPb_2Cl_5$ share planar polyanions with identical geometry laying parallel to the epitaxial interface plane. The same polyanion is shown as seen from above in Figure 3a of the Main Text. In both structures the polyanions are enclosed in a subnetwork of cations, here shown in orange, that is composed of Cs^+ in the case of $CsPb_2Cl_5$ and of Pb^{2+} in the case of $Pb_4S_3Cl_2$. The fact that such subnetwork matches exactly that of $CsPbCl_3$ has a key role in allowing the growth of the heterostructure.

The second comparison highlights the similarities between the cationic subnetworks of $Pb_4S_3Cl_2$ and Cs_2PbCl_4. The different positioning and nature of polyanions causes it to be more compressed in $Pb_4S_3Cl_2$ with respect to Cs_2PbCl_4, but the general distribution of cations, forming cuboidal cages shifted with respect to each other by half lattice step (resulting in the formation of triangular motifs along the projection shown), is identical in both structures.

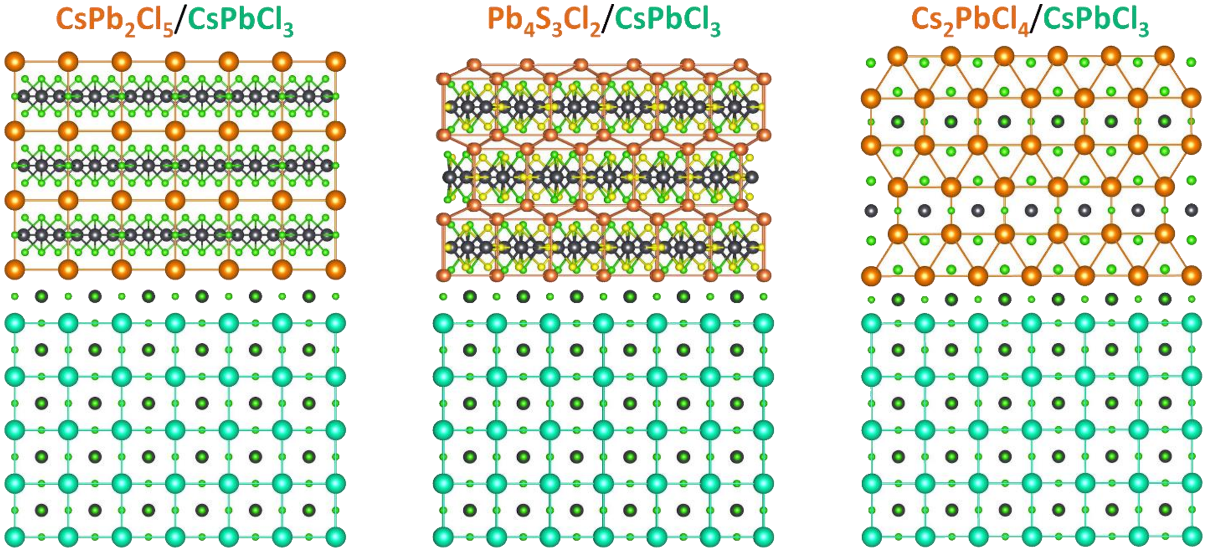

Supplementary Figure 17: comparison of the $Pb_4S_3Cl_2$/$CsPbCl_3$ interface with other hypothetical interfaces. A model of the $Pb_4S_3Cl_2$/$CsPbCl_3$ heterostructure reported in this work is shown in the middle, compared with the hypothetical $CsPb_2Cl_5$/$CsPbCl_3$ (left) and Cs_2PbCl_4/$CsPbCl_3$ heterostructures. Atoms color code: Cs = cyan; Pb = grey; S = yellow; Cl = green. The orange atoms are Pb in $Pb_4S_3Cl_2$ and Cs in $CsPb_2Cl_5$ and Cs_2PbCl_4. This color choice is meant to highlight the similarities between their cationic substructures.

We must note here that we are comparing $Pb_4S_3Cl_2/CsPbCl_3$ with two hypothetical heterostructures because:

I. The $CsPb_2Cl_5/C_sPbCl_3$ interface has never been reported, although there are reports of analogue $CsPb_2Br_5/CsPbBr_3$ heterostructures.[17,18]

II. The Cs_2PbCl_4 structure does not exist, and the closest comparison would be with a $CsPbI_2Cl_2/CsPbCl_3$ heterostructure, as $CsPbI_2Cl_2$ is the only known member of the Cs_2PbX_4 family of compounds. However, although this heterostructure has never been reported, the Cs^+ subnetwork preservation principle we outlined in our previous work on Cs-Pb-X compounds[19] allows to predict with good confidence how it would look like.

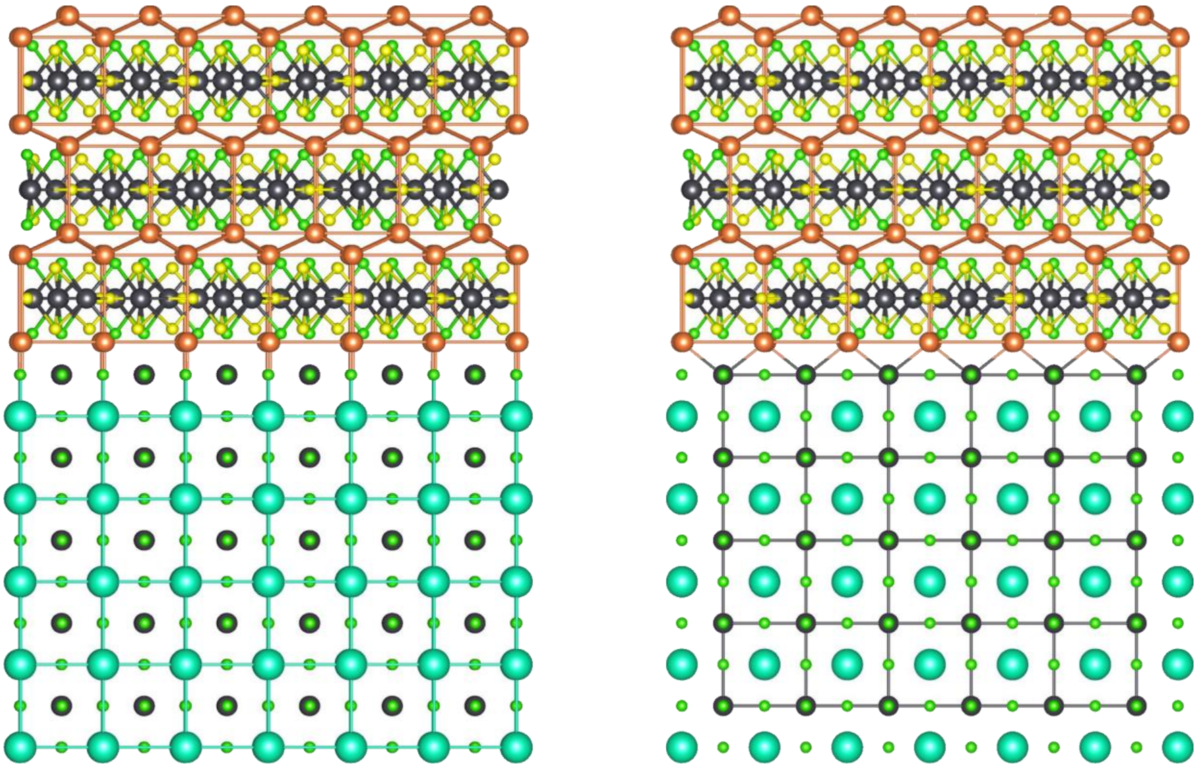

Supplementary Figure 18: Continuity of the cationic subnetworks at the $Pb_4S_3Cl_2/CsPbCl_3$ interface. The Pb^{2+} cationic subnetwork of $Pb_4S_3Cl_2$ finds its prosecution in both the Cs^+ (left) and the Pb^{2+} (right) subnetworks of $CsPbCl_3$ at the same time. The choice of showing the Cs^+ option in the Figure 3a of the Main Text was made to remark the similarities with other Cs-Pb-X/$CsPbX_3$ heterojunctions. Atoms color code: Cs = cyan; Pb = grey/orange; S = yellow; Cl = green.

An atomistic model of the $Pb_4S_3Cl_2/CsPbCl_3$ interface, that can be loaded and visualized with the software VESTA,[14] is provided as a part of the Supplementary Data of this work to help the reader better visualize the structure and the interface connectivity (Supplementary Data 1).

6. Synthesis and characterization of $Pb_4S_3Cl_2/CsPbCl_3$ heterostructures

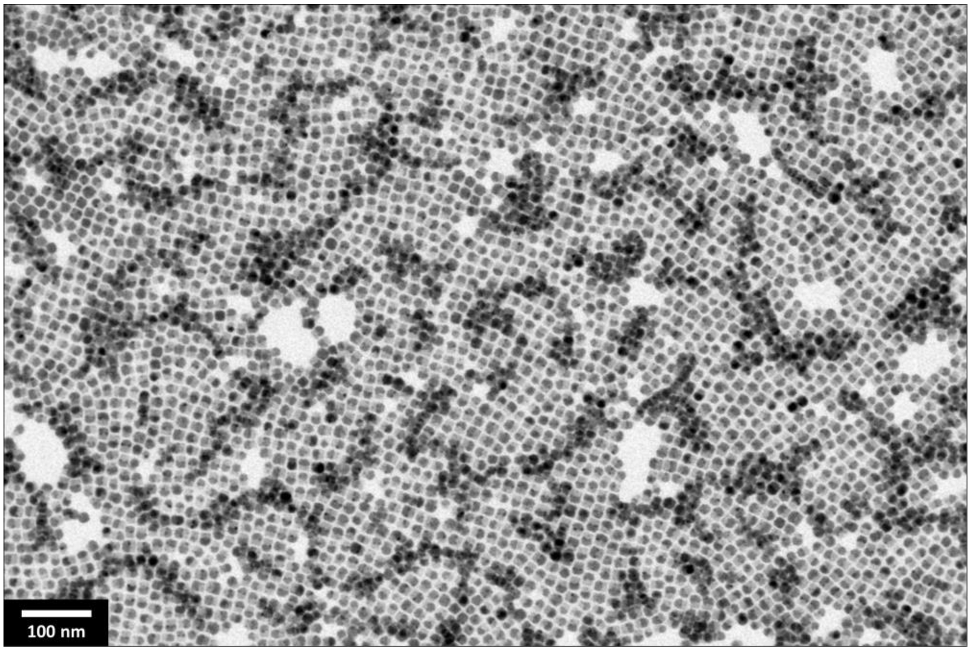

Supplementary Figure 19: TEM image of $Pb_4S_3Cl_2/CsPbCl_3$ heterostructures. The sample was obtained by following the procedure detailed in the Methods section of the Main Text (synthesis + surface treatment).

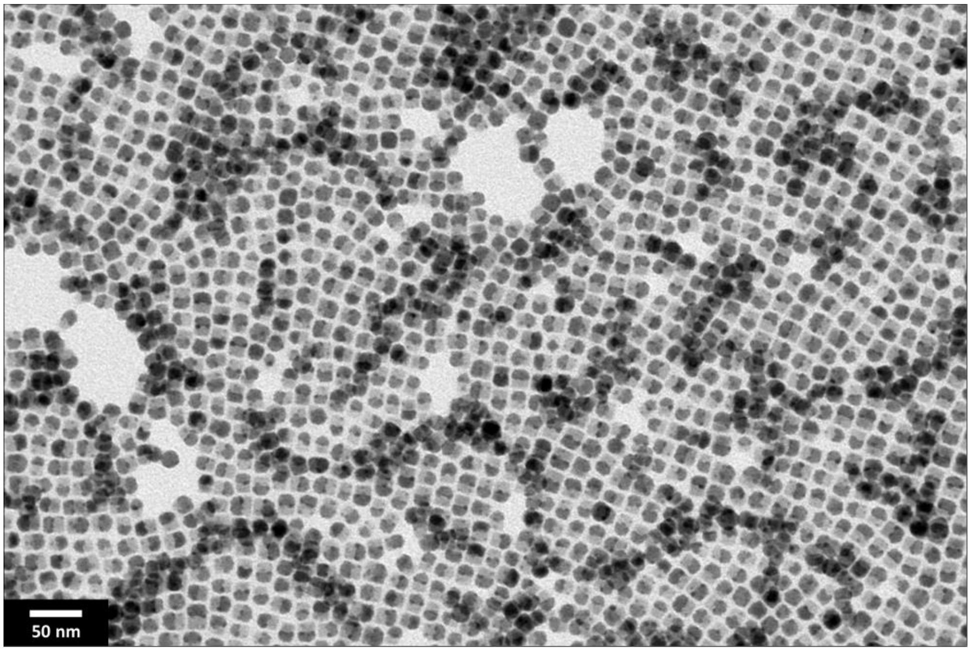

Supplementary Figure 20: TEM image of $Pb_4S_3Cl_2/CsPbCl_3$ heterostructures. Higher magnification image of the same sample shown in the Supplementary Figure 19.

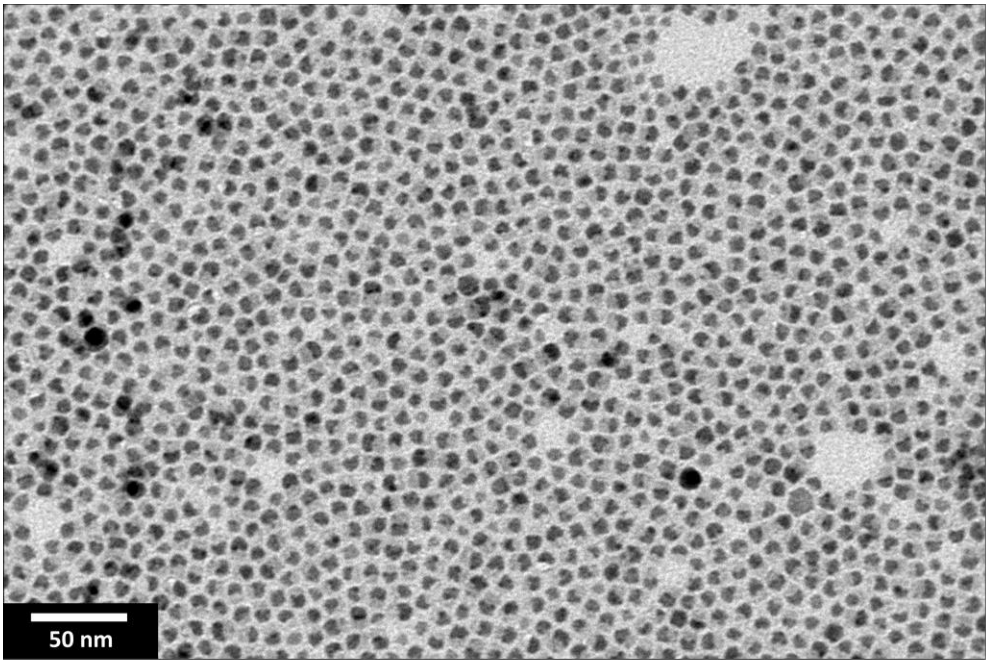

Supplementary Figure 21: TEM image of as-prepared $Pb_4S_3Cl_2/CsPbCl_3$ heterostructures. The sample is shown before the post-synthesis surface treatment.

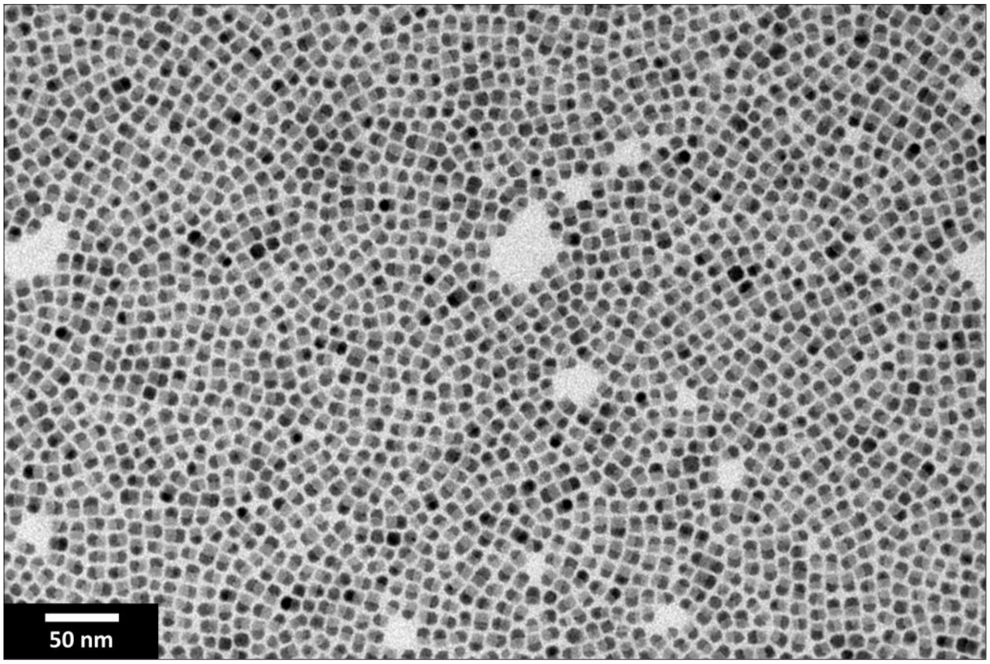

Supplementary Figure 22: TEM image of $Pb_4S_3Cl_2/CsPbCl_3$ heterostructures after the post-synthesis surface treatment. Details on the post-synthesis treatment with a $PbCl_2$ solution can be found in the Methods section of the Main Text. A visual comparison with the Supplementary Figures 19-21 highlights the beneficial influence of such treatment on the homogeneity and morphology of $Pb_4S_3Cl_2/CsPbCl_3$ heterostructures.

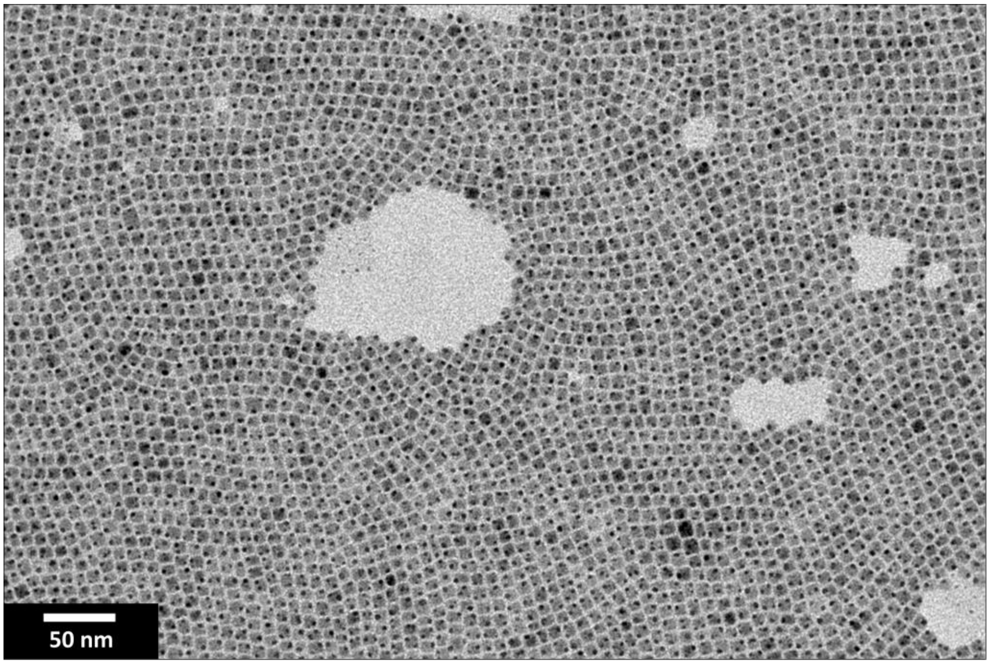

Supplementary Figure 23: TEM image of $CsPbCl_3$ NCs. The sample was obtained by heating at 200°C a batch of $CsPbCl_3$ nanoclusters dissolved in 1-octadecene in the absence of elemental sulfur, dodecanethiol and lead oleate.

7. Etching of heterostructures and characterization of $Pb_4S_3Cl_2$ nanocrystals

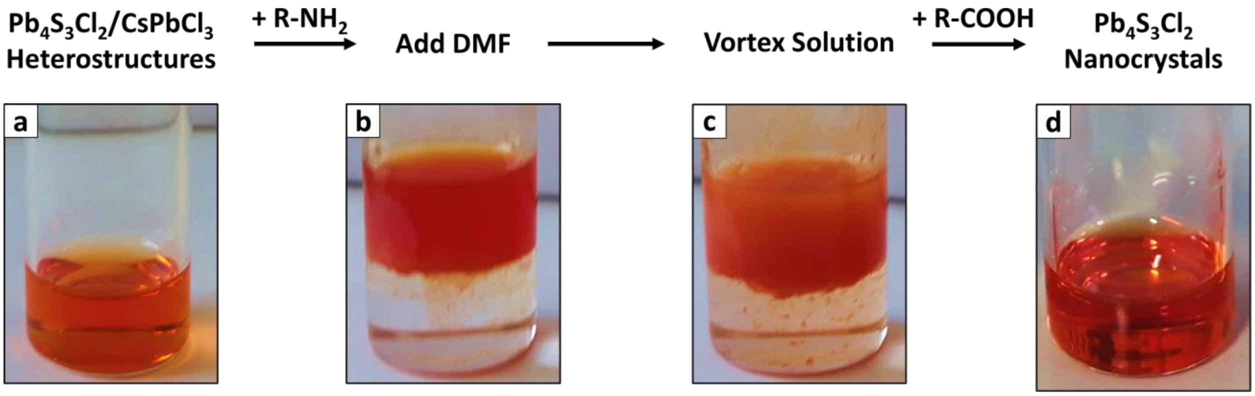

Supplementary Figure 24: Colloidal dispersions of $Pb_4S_3Cl_2/CsPbCl_3$ heterostructures at various stages of the etching process. From left to right: a) initial heterostructures solution; b) following the addition of oleylamine (R-NH_2) and dimethylformamide (DMF); c) after vortexing and decanting the mixture; d) after the addition of oleic acid (R-COOH) and the removal of DMF. The etching treatment was performed as detailed in the Methods section of the Main Text.

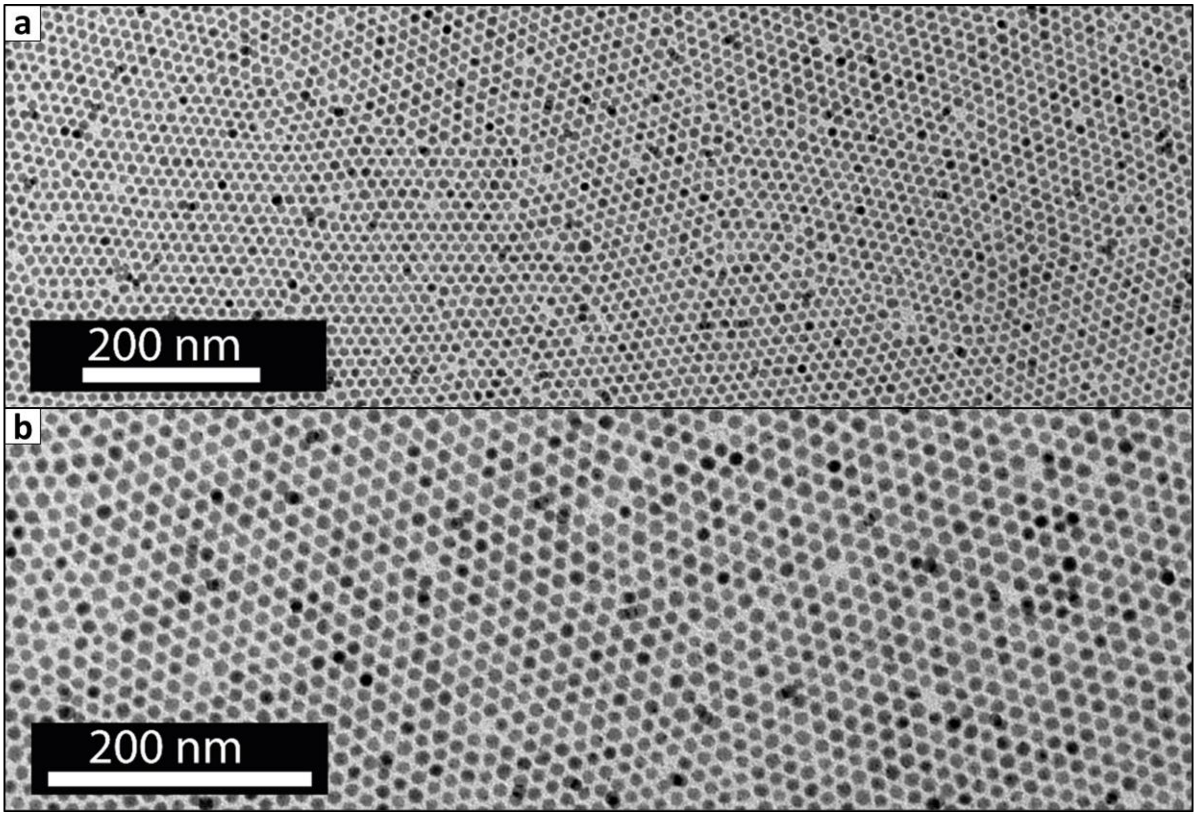

Supplementary Figure 25: TEM images of $Pb_4S_3Cl_2$ NCs obtained by etching the $CsPbCl_3$ domains off the $Pb_4S_3Cl_2/CsPbCl_3$ heterostructures. The two panels are shown at different magnifications.

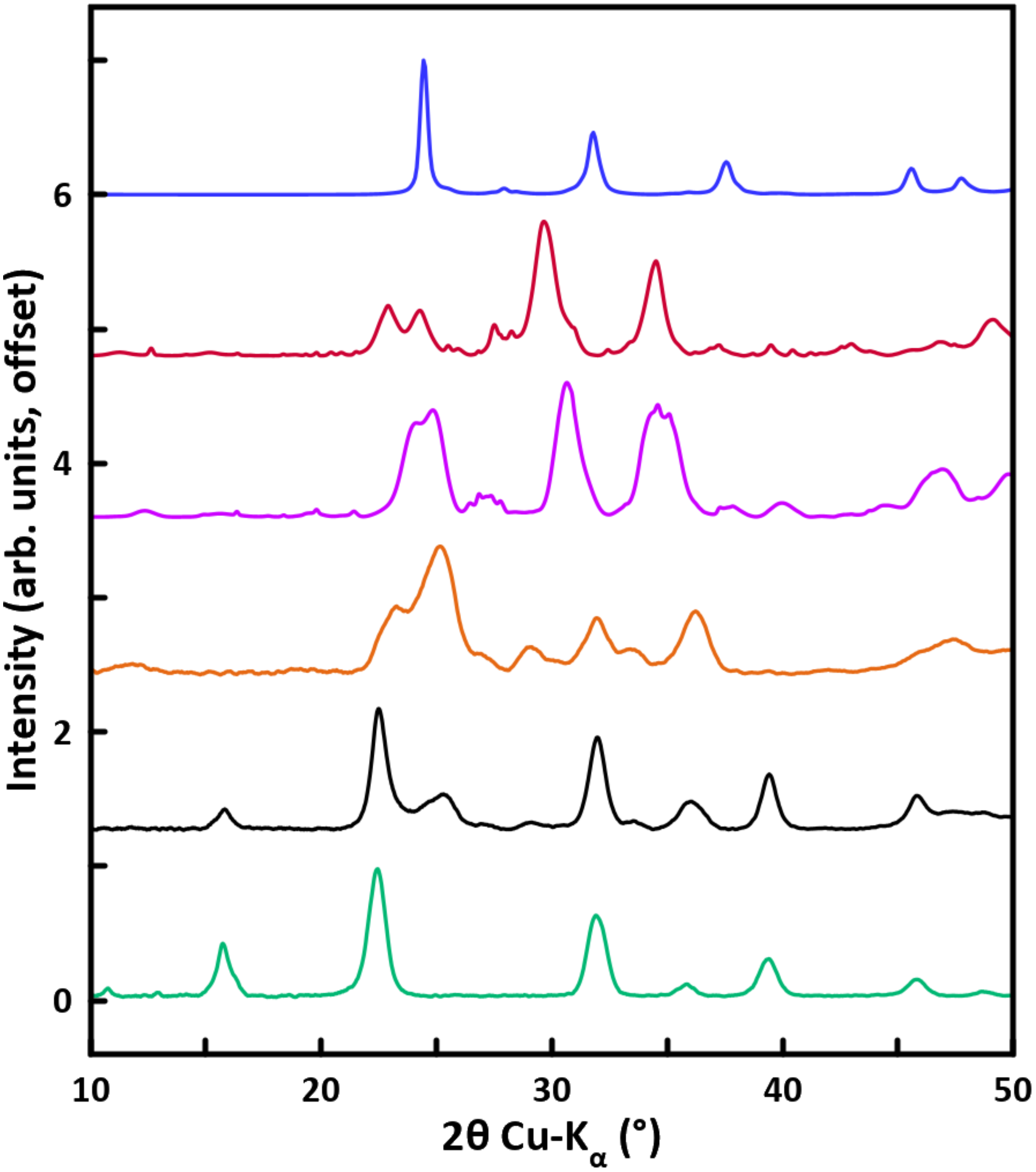

Supplementary Figure 26: XRPD patterns of $Pb_3S_2Cl_2$, $Pb_4S_3I_2$, $Pb_4S_3Br_2$, $Pb_4S_3Cl_2$, $CsPbCl_3$ NCs, and $Pb_4S_3Cl_2/CsPbCl_3$ heterostructures. The three $Pb_4S_3X_2$ NCs patterns feature strong similarities in terms of general distribution and intensity of diffraction peaks, further confirming the that the NCs obtained by etching the heterostructures retain the $Pb_4S_3Cl_2$ structure. However, a more elaborate analysis of the diffractogram is hindered by the extreme peak broadening and the overall low quality. To improve the data visualization, a moving-average smoothing algorithm was applied to some of the patterns ($Pb_4S_3Cl_2$, $CsPbCl_3$, heterostructures). Color code: $Pb_4S_3Cl_2$ = blue, $Pb_4S_3I_2$ = red, $Pb_4S_3Br_2$ = purple, $Pb_4S_3Cl_2$ = orange, $Pb_4S_3Cl_2/CsPbCl_3$ heterostructures = black, $CsPbCl_3$ = cyan. Source data are provided as a Source Data file.

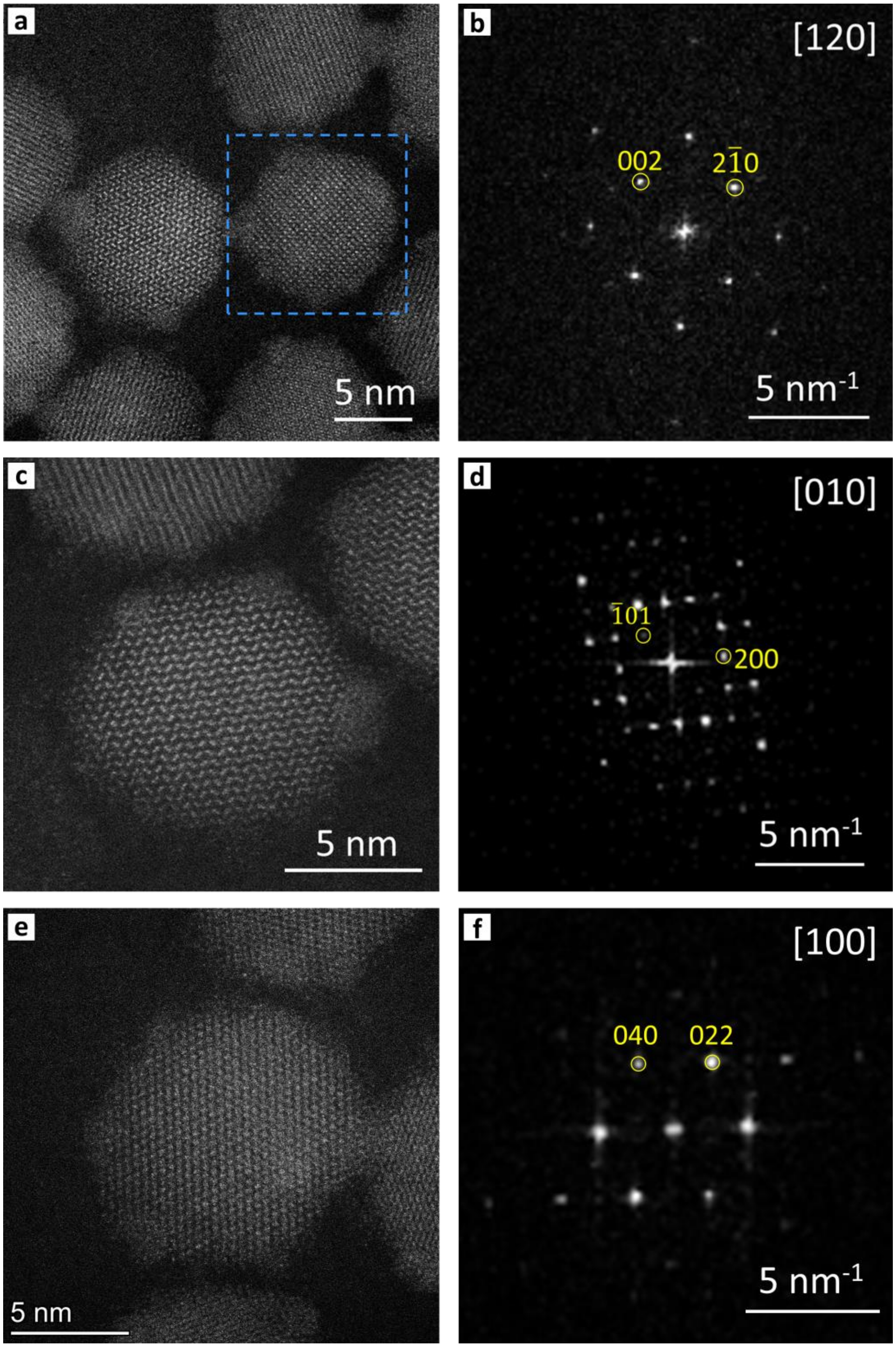

Supplementary Figure 27: HAADF-STEM characterization of free-standing $Pb_4S_3Cl_2$ NCs. The sample was obtained by etching the $Pb_4S_3Cl_2$/$CsPbCl_3$ heterostructures. The panels on the left (a,c,e,) show high-resolution HAADF-STEM images of colloidal $Pb_4S_3Cl_2$ NCs, while the panels on the right (b,d,f) show the corresponding FFTs. In all the cases, the FFTs could be indexed according to the $Pb_4S_3Cl_2$ structure, confirming that the NCs phase is retained during the etching process.

8. Spectroscopic characterization

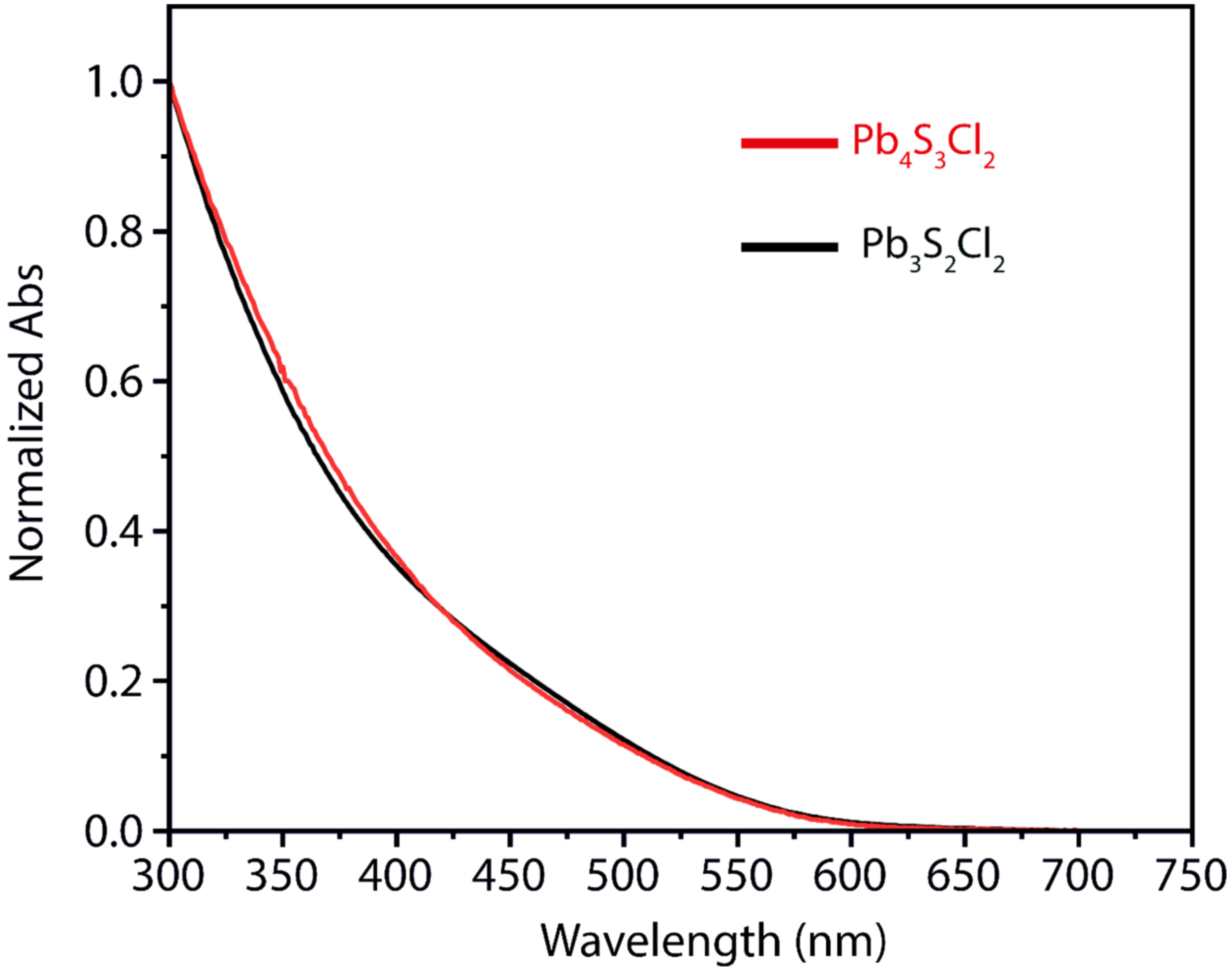

Supplementary Figure 28: Absorption spectra of free-standing lead sulfochloride NCs. Optical absorption spectra of $Pb_3S_2Cl_2$ NCs (black solid line) and $Pb_4S_3Cl_2$ NCs (red solid line) normalized and compared. The latter were obtained by selectively etching the $CsPbCl_3$ domain from the $Pb_4S_3Cl_2$/$CsPbCl_3$ heterostructures. Source data are provided as a Source Data file.

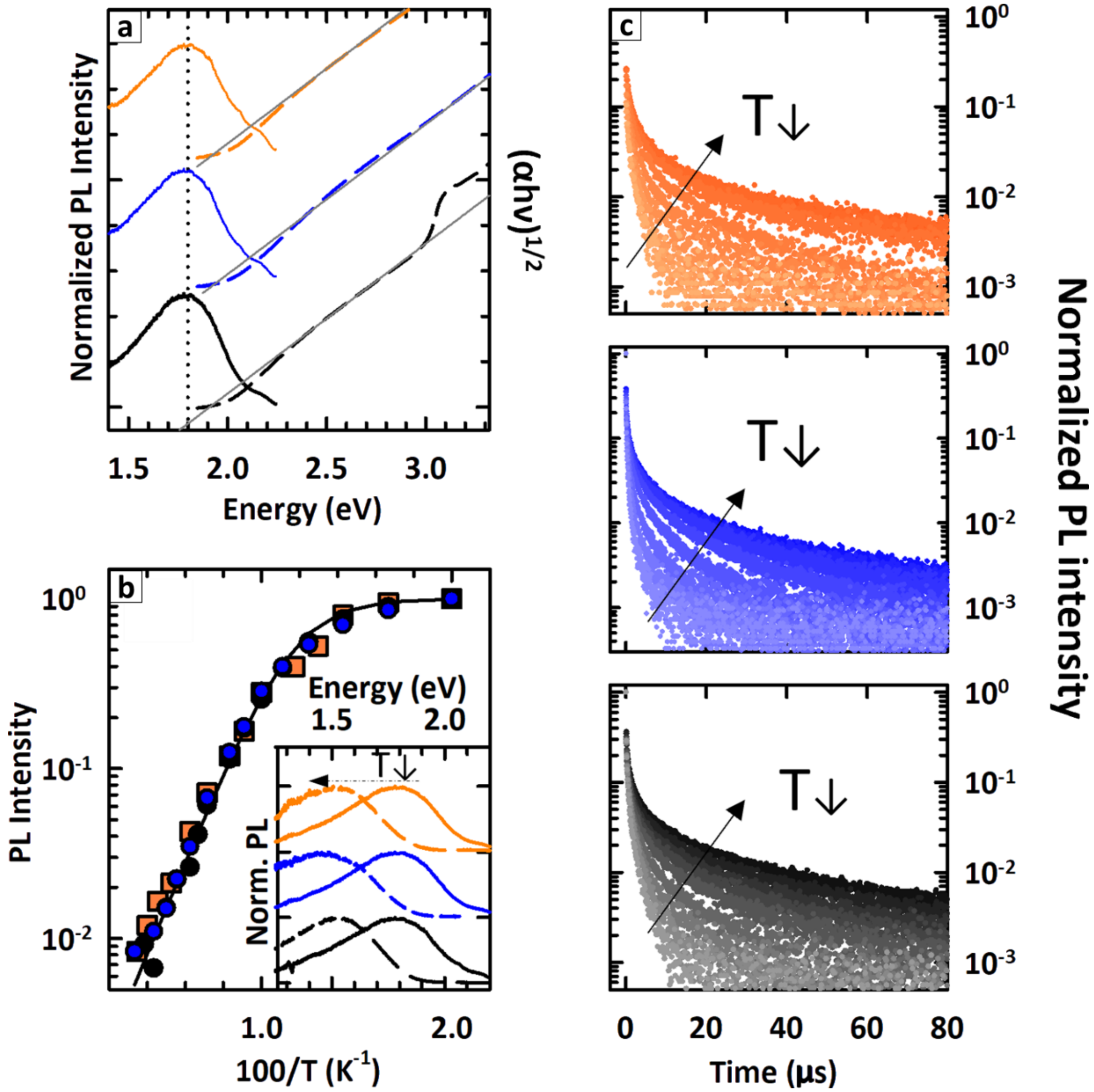

Supplementary Figure 29: Spectroscopic characterizations of lead sulfochloride NCs and $Pb_4S_3Cl_2/CsPbCl_3$ heterostructures. a) Photoluminescence spectra (solid lines) and Tauc plot analysis (dashed lines) of the absorption spectra of $Pb_3S_2Cl_2$ NCs (blue), $Pb_4S_3Cl_2$ NCs (orange) and $Pb_4S_3Cl_2/CsPbCl_3$ heterostructures (black), superimposed to the corresponding fit lines (grey). The analysis highlights that all the three materials are characterized by comparable band gaps and emissions. b) Temperature dependence of the photoluminescence intensity of $Pb_3S_2Cl_2$ NCs (blue), $Pb_4S_3Cl_2$ NCs (orange) and $Pb_4S_3Cl_2/CsPbCl_3$ heterostructures (black). Inset: temperature-dependent spectral shift of the photoluminescence spectra, measured at 300K (solid lines) and at 77K (dashed lines). c) Time resolved photoluminescence spectra as a function of temperature for the three samples. Darker lines indicate lower temperatures, lighter lines higher temperatures. Source data are provided as a Source Data file.

Supplementary References

1. Schindelin, J. *et al.* Fiji: An open-source platform for biological-image analysis. *Nature Methods* vol. 9 676–682 (2012).
2. Park, S. D. *et al.* Bandgap Inhomogeneity of a PbSe Quantum Dot Ensemble from Two-Dimensional Spectroscopy and Comparison to Size Inhomogeneity from Electron Microscopy. *Nano Lett.* **17**, 762–771 (2017).
3. Yun, Y., Zou, X., Hovmöller, S. & Wan, W. Three-dimensional electron diffraction as a complementary technique to powder X-ray diffraction for phase identification and structure solution of powders. *IUCrJ* **2**, 267–282 (2015).
4. Gemmi, M. *et al.* 3D Electron Diffraction: The Nanocrystallography Revolution. *ACS Cent. Sci.* **5**, 1315–1329 (2019).
5. Vincent, R. & Midgley, P. A. Double conical beam-rocking system for measurement of integrated electron diffraction intensities. *Ultramicroscopy* **53**, 271–282 (1994).
6. Mugnaioli, E., Gorelik, T. & Kolb, U. 'Ab initio' structure solution from electron diffraction data obtained by a combination of automated diffraction tomography and precession technique. *Ultramicroscopy* **109**, 758–765 (2009).
7. Kolb, U., Krysiak, Y. & Plana-Ruiz, S. Automated electron diffraction tomography - Development and applications. *Acta Crystallogr. Sect. B Struct. Sci. Cryst. Eng. Mater.* **75**, 463–474 (2019).
8. Burla, M. C. *et al.* Crystal structure determination and refinement via SIR2014. *J. Appl. Crystallogr.* **48**, 306–309 (2015).
9. Ni, D., Guo, S., Powderly, K. M., Zhong, R. & Cava, R. J. A high-pressure phase with a non-centrosymmetric crystal structure in the PbSe–$PbBr_2$ system. *J. Solid State Chem.* **280**, (2019).
10. Altomare, A. *et al.* EXPO2013: A kit of tools for phasing crystal structures from powder data. *J. Appl. Crystallogr.* **46**, 1231–1235 (2013).
11. Farrow, C. L. *et al.* PDFfit2 and PDFgui: Computer Programs for Studying Nanostructure in Crystals. *J. Phys. Condens. Matter* **19**, 335219 (2007).
12. Caliandro, R. & Belviso, D. B. RootProf: Software for Multivariate Analysis of Unidimensional Profiles. *J. Appl. Crystallogr.* **47**, 1087–1096 (2014).
13. Juhás, P., Farrow, C. L., Yang, X., Knox, K. R. & Billinge, S. J. L. Complex Modeling: a Strategy and Software Program for Combining Multiple Information Sources to Solve Ill posed Structure and Nanostructure Inverse Problems. *Acta Crystallogr. Sect. A Found. Adv.* **71**, 562–568 (2015).
14. Momma, K. & Izumi, F. VESTA: A three-dimensional visualization system for electronic and structural analysis. *J. Appl. Crystallogr.* **41**, 653–658 (2008).
15. Imran, M. *et al.* Halide Perovskite-Lead Chalcohalide Nanocrystal Heterostructures. *J. Am. Chem. Soc.* **143**, 1435–1446 (2021).

16. Toso, S. *et al.* Nanocrystals of Lead Chalcohalides: A Series of Kinetically Trapped Metastable Nanostructures. *J. Am. Chem. Soc.* **142**, 10198–10211 (2020).
17. Zheng, Y. *et al.* All-inorganic dual-phase halide perovskite nanorings. *Nano Res.* **13**, 2994–3000 (2020).
18. Huang, Z. P. *et al.* In Situ Growth of 3D/2D ($CsPbBr_3$/$CsPb_2Br_5$) Perovskite Heterojunctions toward Optoelectronic Devices. *J. Phys. Chem. Lett.* **11**, 6007–6015 (2020).
19. Toso, S., Baranov, D. & Manna, L. Hidden in Plain Sight: The Overlooked Influence of the Cs^+ Substructure on Transformations in Cesium Lead Halide Nanocrystals. *ACS Energy Lett.* 3409–3414 (2020) doi:10.1021/acsenergylett.0c02029.
